# Selective Doping to Controllably Tailor Maximum Unit‐Cell‐Volume Change of Intercalating Li^+^‐Storage Materials: A Case Study of *γ* Phase Li_3_VO_4_

**DOI:** 10.1002/advs.202106003

**Published:** 2022-06-24

**Authors:** Jianbin Deng, Changpeng Lv, Tian Jiang, Siyuan Ma, Xuehua Liu, Chunfu Lin

**Affiliations:** ^1^ Institute of Materials for Energy and Environment School of Materials Science and Engineering Qingdao University Qingdao 266071 China

**Keywords:** high‐temperature operation, in situ characterization, intercalating Li^+^‐storage material, selective doping, zero‐strain

## Abstract

Capacity decay of an intercalating Li^+^‐storage material is mainly due to the its particle microcracks from stress‐causing volume change. To extend its cycle life, its unit‐cell‐volume change has to be minimized as much as possible. Here, based on a *γ*‐Li_3_VO_4_ model material, the authors explore selective doping as a general strategy to controllably tailor its maximum unit‐cell‐volume change, then clarify the relationship between its crystal‐structure openness and maximum unit‐cell‐volume change, and finally demonstrate the superiority of “zero‐strain” materials within 25–60 °C (especially at 60 °C). With increasing the large‐sized Ge^4+^ dopant, the unit‐cell volume of *γ*‐Li_3+_ *_x_* Ge*_x_* V_1−_ *_x_* O_4_ becomes larger and its crystal structure becomes looser, resulting in the decrease of its maximum unit‐cell‐volume change. In contrast, the doping with small‐sized Si^4+^ shows a reverse trend. The tailoring reveals that *γ*‐Li_3.09_Ge_0.09_V_0.91_O_4_ owns the smallest maximum unit‐cell‐volume change of 0.016% in the research field of intercalating Li^+^‐storage materials. Consequently, *γ*‐Li_3.09_Ge_0.09_V_0.91_O_4_ nanowires exhibit excellent cycling stability at 25/60 °C with 94.8%/111.5% capacity‐retention percentages after 1800/1500 cycles at 2 A g^−1^. This material further shows large reversible capacities, proper working potentials, and high rate capability at both temperatures, fully demonstrating its great application potential in long‐life lithium‐ion batteries.

## Graphical abstract

Selective doping is explored as a general strategy to controllably tailor the maximum unit‐cell‐volume change of intercalating Li^+^‐storage materials. The tailoring follows the law of doping with large‐sized ions → increasing unit‐cell volume (i.e., increasing crystal‐structure openness) → decreasing maximum unit‐cell‐volume change. Additionally, it is demonstrated that the “zero‐strain” characteristic is greatly beneficial for the cycling stability at high temperatures.

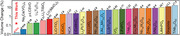

## 1 Introduction

Ultralong cycle life is highly pursued for lithium‐ion batteries (LIBs) because the use cost of LIBs in not only small‐scale portable electronics but also large‐scale energy‐storage applications can be significantly decreased.^[^ ^1^ ^]^ The commercial LIBs generally use intercalating Li^+^‐storage materials, which suffer from unit‐cell‐volume change (such as T‐Nb_2_O_5_) and/or undergo two‐phase transition (such as LiFePO_4_) during Li^+^ insertion.^[^ ^1e^ ^]^ The volume changes in both cases are usually obvious and highly reversible during Li^+^ extraction. During the repeated Li^+^ insertion–extraction, the accumulative stress arising from the unit‐cell‐volume change and/or mismatched lattice constants between the two phases becomes significant, causing the microcracks of active‐material particles. These microcracks gradually break the active‐material particles, then deteriorate the electrode mechanical integrity (i.e., disconnect the Li^+^/electron transport pathways), and eventually cause the Li^+^‐storage capacity decay.^[^ ^2^ ^]^ For instance, it is confirmed that the fast capacity decay of the popular nickel‐rich LiNi_1−_ *_x_*_−_ *_y_* Co*_x_* Mn*_y_* O_2_ and LiNi_1−_ *_x_*_−_ *_y_* Co*_x_* Al*_y_* O_2_ cathode materials is rooted in the microcracks from stress‐causing anisotropic change.^[^ ^3^ ^]^ Therefore, the unit‐cell‐volume changes of intercalating Li^+^‐storage materials have to be minimized as much as possible to extend their cycle life.

In this regard, “zero‐strain” Li^+^‐storage materials with tiny unit‐cell‐volume change of <1% have attracted intensive research interest.^[^ ^2^, ^4^ ^]^ The very small volume variation generates only small stress within the “zero‐strain” Li^+^‐storage material particles. Consequently, the microcrack formation can be avoided and good cycling stability can be achieved. For instance, the excellent cycling stability of Li_4_Ti_5_O_12_ for thousands of repeated cycles is undoubtedly originated from its “zero‐strain” characteristic with only ≈0.2% unit‐cell‐volume change during Li^+^ insertion–extraction.^[^ ^4a^ ^]^ However, the reported “zero‐strain” Li^+^‐storage materials (Li_4_Ti_5_O_12_, NbO_2_, LiY(MoO_4_)_3_, Na_2_Ca(VO_3_)_4_, *γ*‐Li_3.08_Cr_0.02_Si_0.09_V_0.9_O_4_, LiCrTO_4_, Li_2_Ni_0.2_Co_0.18_O_4_, LiRh_2_O_4_, and LiCaFeF_6_) are very limited.^[^ ^4^ ^]^ Apparently, the strategy that the unit‐cell‐volume change of a Li^+^‐storage material is tailored to achieve zero‐strain is highly desirable for its ultralong operation, but few works on this topic have been reported so far. Here, we expect that such tailoring can be achieved through proper doping of the Li^+^‐storage material based on a previous report showing that doping affected the unit‐cell‐volume change of LiFePO_4_.^[^ ^5^ ^]^

It is known that both energy and power densities of LIBs rapidly decrease at elevated temperatures,^[^ ^6^ ^]^ limiting the use of LIBs in hot situations (such as summer periods and tropical regions). At high temperatures, the excessive Li^+^ insertion into intercalating Li^+^‐storage materials (such as graphite and TiO_2_) induces overly large stress in their significantly expanded unit cells, ultimately causing serious particle crack and fast capacity decay.^[^ ^7^ ^]^ It is great challenge to tackle this problem of high‐temperature performance decay. Here, we expect that an intercalating Li^+^‐storage material with a “zero‐strain” characteristic should have good high‐temperature cycling stability since its unit‐cell‐volume change is insensitive to the inserted Li^+^ amount.

In this work, we first explore selective doping as a general strategy to controllably tailor the maximum unit‐cell‐volume change of an intercalating Li^+^‐storage material, then clarify the relationship between its crystal‐structure openness and maximum unit‐cell‐volume change, and finally demonstrate the superiority of “zero‐strain” materials in a broad temperature range of 25–60 °C (specially at high temperatures). *γ*‐Li_3_VO_4_ is selected as the model material. Various Ge^4+^ doped *γ*‐Li_3_VO_4_ (*γ*‐Li_3+_ *_x_* Ge*_x_* V_1−_ *_x_* O_4_, *x* = 0.06, 0.08, 0.09, and 0.10) compounds are prepared through solid‐state reaction. With increasing the Ge^4+^ dopant, the unit‐cell volume of *γ*‐Li_3+_ *_x_* Ge*_x_* V_1−_ *_x_* O_4_ becomes larger and its crystal structure becomes looser, leading to the decrease of its maximum unit‐cell‐volume change. In stark contrast, the doping of *γ*‐Li_3_VO_4_ with small‐sized Si^4+^ reveals a reverse trend. Through the crystal‐structure tailoring, it is found that *γ*‐Li_3.09_Ge_0.09_V_0.91_O_4_ (*γ*‐LGVO‐0.09) shows the smallest maximum unit‐cell‐volume change (only 0.016%) among the reported intercalating Li^+^‐storage materials. Therefore, *γ*‐Li_3.09_Ge_0.09_V_0.91_O_4_ nanowires (*γ*‐LGVO‐0.09‐NW) synthesized through electrospinning exhibit excellent cycling stability (capacity‐retention percentage of 95.2% after 1500 cycles at 2 A g^−1^). This material further shows a proper working potential, high rate capability and large reversible capacity (383 mAh g^−1^). Attractively, at the high temperature of 60 °C, it retains excellent cycling stability (capacity‐retention percentages of 111.5% and 103.7% after 1500 cycles at 2 and 4 A g^−1^, respectively), and enhances the rate capability and reversible capability (425 mAh g^−1^).

## 2 Results and Discussion

**Figure 1:**
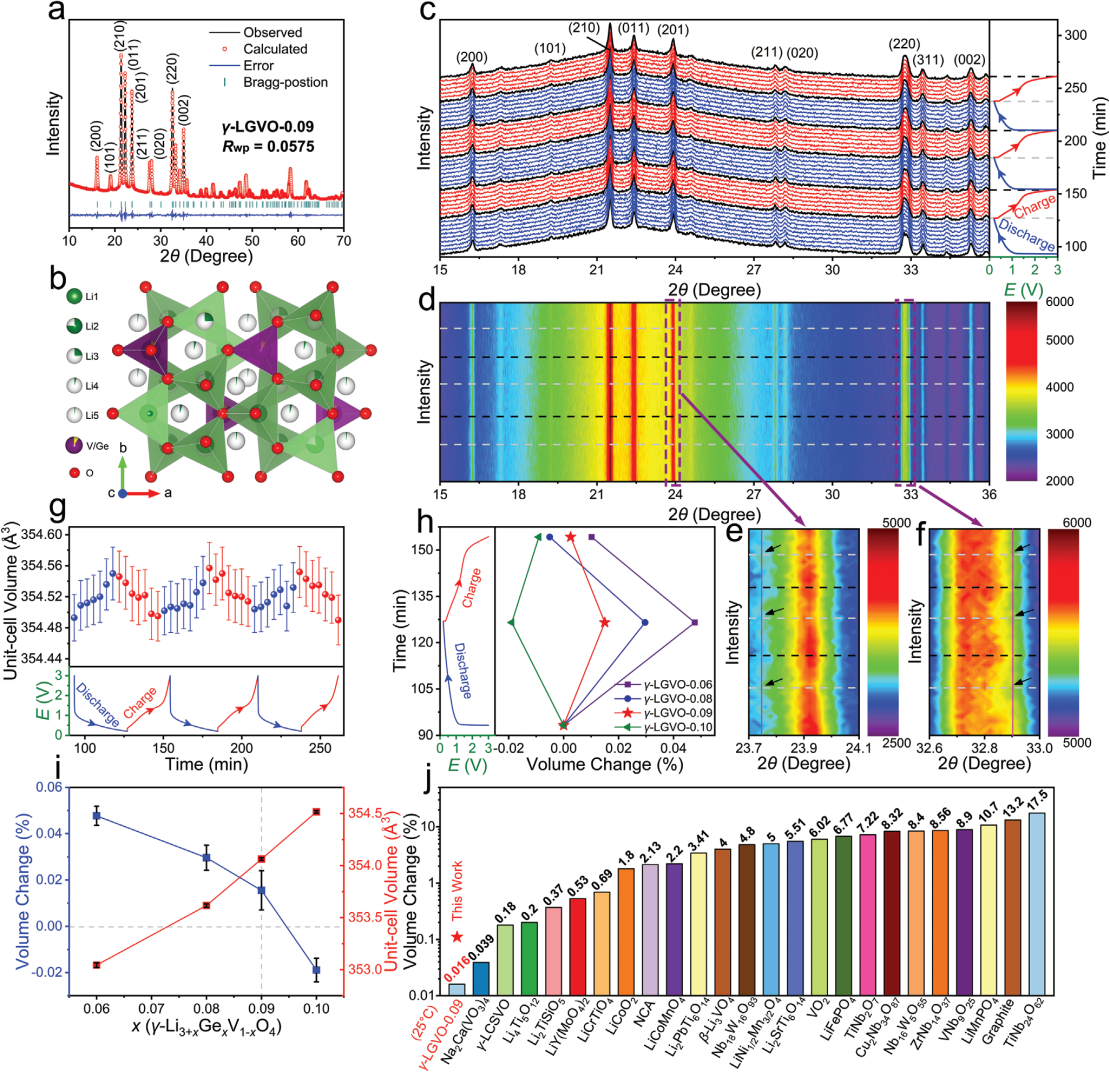
Crystal structure of *γ*‐LGVO‐0.09. a) XRD pattern with Rietveld refinement (main peaks are labeled) and b) schematic crystal structure of *γ*‐LGVO‐0.09. c) Pristine and d) 2D in situ XRD patterns of *γ*‐LGVO‐0.09 with corresponding GCD curves after initial cycle within 3.0–0.2 V at 0.5 A g^−1^. Enlarged e) (201) and f) (220) peaks in d). Peak shifts are highlighted by black arrows. g) Variation in unit‐cell volume of *γ*‐LGVO‐0.09. h) Variations in unit‐cell volume during discharge/charge and i) maximum unit‐cell‐volume‐change percentages and unit‐cell volumes of *γ*‐LGVO‐*x* (*x* = 0.06, 0.08, 0.09, and 0.10) samples. Error bar represents one standard deviation of uncertainty. j) Maximum unit‐cell‐volume change of *γ*‐LGVO‐0.09 compared with intercalating Li^+^‐storage materials previously reported (*γ*‐LCSVO and NCA represent *γ*‐Li_3.08_Cr_0.02_Si_0.09_V_0.9_O_4_ and LiNi_0.8_Co_0.15_Al_0.05_O_2_, respectively).

The Rietveld‐refined X‐ray diffraction (XRD) patterns of *γ*‐LGVO‐0.09, *γ*‐LGVO‐*x*, and *γ*‐LSVO‐*x* (*x* = 0.06, 0.08, and 0.10) are illustrated in **Figure** **1**a; and Figure S1 (Supporting Information),^[^ ^8^ ^]^ and their lattice constants and the fractional atomic parameters of *γ*‐LGVO‐0.09 obtained from the refinements are shown in Tables S1 and S2 (Supporting Information), respectively. All the Rietveld‐refined XRD patterns are in good agreement with their experimental patterns with reasonable weighted profile residual factors (*R*_wp_), indicating a series of successful dopings in the crystal lattice of *γ*‐Li_3_VO_4_. The characteristic diffraction peaks of *γ*‐LGVO‐0.09 at 16.07°, 19.10°, 21.36°, 22.22°, 23.77°, 27.63°, 28.03°, 32.55°, and 35.01° correspond to the (200), (101), (210), (011), (201), (211), (020), (220), and (002) crystallographic planes. The crystal structure of *γ*‐LGVO‐0.09 (Figure 1b) matches with an orthogonal system with *Pnma* space group, which is composed of isolated (V/Ge)O_4_ tetrahedra fully surrounded by LiO_4_ tetrahedra and LiO_5_ trigonal bipyramids.^[^ ^9^ ^]^ Each (V/Ge)O_4_ tetrahedron corner‐shares with two LiO_4_ tetrahedra and six LiO_5_ trigonal bipyramids, and edge‐shares with another two LiO_5_ trigonal bipyramids. The LiO_4_ tetrahedra and LiO_5_ trigonal bipyramids are connected through corner and/or edge sharing, forming an ordered network, in which (V/Ge)O_4_ tetrahedra act as a portion of network nodes and plenty of wide Li^+^ transport pathways along the *c* axis are generated for fast Li^+^ transport and storage. Therefore, *γ*‐LGVO‐0.09 is a fast Li^+^ conductor, similar to the famous *γ*‐Li_3_PO_4_ solid electrolyte with the same type of crystal structure.^[^ ^10^ ^]^ Based on the *β → γ* phase transformation mechanism of Li_3_VO_4_,^[^ ^11^ ^]^ a certain amount of Ge^4+^ and Li^+^ ions are selected to replace partial V^5+^ ions based on the defect equation $2{\mathrm{GeO}}_{2}+{\mathrm{Li}}_{2}O\;=2{\mathrm{Ge}}_{V}^{,}\;+2{\mathrm{Li}}_{i}^{\cdot }+5O_{O}^{\times }$, which enables the stabilization of the *γ*‐Li_3_VO_4_ high‐temperature phase at the room temperature. The additional Li^+^ ions insert into Li(4) and Li(5) interstitial sites, rendering a portion of Li^+^ ions in Li(2) sites migrate to neighboring Li(3) sites.^[^ ^9^ ^]^ Besides the Ge^4+^ doping, the Si^4+^ doping is also successful based on the defect equation $2{\mathrm{SiO}}_{2}+{\mathrm{Li}}_{2}O\;=2{\mathrm{Si}}_{V}^{,}\;+2{\mathrm{Li}}_{i}^{\cdot }+5O_{O}^{\times }$.^[^ ^11^ ^]^ Interestingly, the Si^4+^ doping decreases the unit‐cell volume of *γ*‐LSVO‐*x*, whereas the unit‐cell volume of *γ*‐LGVO‐*x* increases with the Ge^4+^ amount (Table S1, Supporting Information), which is undoubtedly due to the much larger ion size of Ge^4+^ (0.39 Å) than that of Si^4+^ (0.26 Å) in the tetrahedral coordination.^[^ ^12^ ^]^

In situ XRD tests of *γ*‐LGVO‐0.09/Li, *γ*‐LGVO‐*x*/Li, and *γ*‐LSVO‐*x*/Li half cells (*x* = 0.06, 0.08, and 0.10) were conducted to reveal the crystal‐structure evolution based on quantitative Ge^4+^/Si^4+^ dopings in *γ*‐Li_3_VO_4_ (Figure 1c,d; and Figure S2, Supporting Information).^[^ ^13^ ^]^ The in situ XRD patterns after the initial activation cycle show high reversibility during Li^+^ insertion–extraction processes where all the characteristic peaks negligibly change their peak positions and intensities, as highlighted in Figure 1e,f, indicating the “zero‐strain” characteristic of these *γ*‐Li_3_VO_4_‐based materials. The exact lattice‐constant variations of each sample were yielded using Rietveld refinements of the in situ XRD patterns at different discharge/charge states (Figure 1g,h; and Figure S3, Supporting Information). With the increase of the Si^4+^ amount in *γ*‐Li_3_VO_4_, the change percentage of the unit‐cell volume upon Li^+^ insertion becomes larger (Figure S4, Supporting Information), while the case of the Ge^4+^ doping shows an inverse trend (Figure 1i). This interesting phenomenon can be well explained by the different volume‐buffering capability of the doped *γ*‐Li_3_VO_4_ materials upon Li^+^ insertion. For Si^4+^ doped *γ*‐Li_3_VO_4_, the unit‐cell volume of *γ*‐LSVO‐*x* monotonously decreases with the Si^4+^ amount (Figure S4, Supporting Information). This more compact crystal structure undoubtedly decreases the volume‐buffering capability, leading to the increase of the volume‐change percentage. In stark contrast, the structure of Ge^4+^ doped *γ*‐Li_3_VO_4_ becomes looser when increasing the Ge^4+^ amount (Figure 1i), resulting in the enhancement of the volume‐buffering capability and the decrease of the volume‐change percentage.

The variation of the volume‐change percentage increases with the doping amount. Apparently, large variations of the volume‐change percentage can be achieved in the heavy doping and substitution cases. For instance, based on J. Shu's two reports,^[^ ^14^ ^]^ VNb_9_O_25_ shows a significantly smaller maximum unit‐cell‐volume change (8.91%) than isostructural PNb_9_O_25_ (10.69%) upon Li^+^ insertion since V^5+^ has a much larger ion size (0.355 Å) than P^5+^ (0.17 Å) in the tetrahedral coordination. Similar situations happen in another three cases of Li(Fe_0.875_Zr_0.125_)(P_0.75_Si_0.25_)O_4_ versus LiFePO_4_, LiCrTiO_4_ versus Li_4_Ti_5_O_12_ versus LiTi_2_O_4_, and Ni_2_Nb_34_O_87_ versus GaNb_11_O_29_ (see details in the Supporting Information).^[^ ^5^, ^15^ ^]^ Therefore, the relationship between the crystal‐structure openness and the maximum unit‐cell‐volume change we found in this work is general in the research field of intercalating Li^+^‐storage materials. The unit‐cell volume of *γ*‐LGVO‐0.09 varies from 354.493 Å^3^ (full delithiation) to 354.546 Å^3^ (full lithiation), achieving the smallest volume‐change percentage of 0.016% among all the known Li^+^‐storage materials (Figure 1j; and Table S3, Supporting Information). It is noteworthy that the volume‐change percentage becomes negative when 10% of Ge^4+^ is doped (Figure 1i), suggesting that the real zero volume‐change can be achieved at an *x* value between 0.09 and 0.10 for *γ*‐Li_3+_ *_x_* Ge*_x_* V_1−_ *_x_* O_4_. However, we did not try to obtain the exact *x* value for the real zero volume‐change since the precision of our in situ XRD experiments and Rietveld refinements is insufficient to achieve this attractive target.

**Figure 2:**
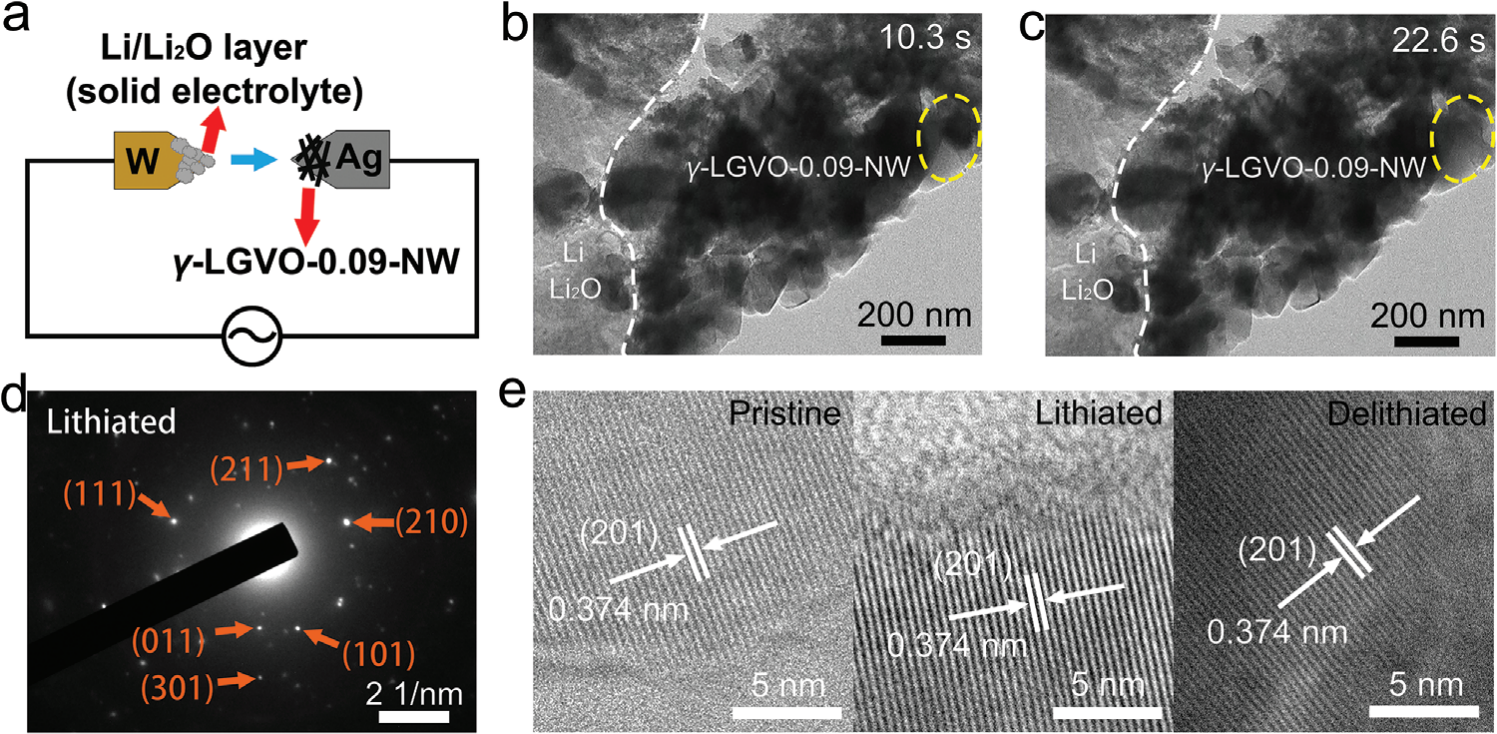
In situ and ex situ TEM characterizations. a) Schematic illustration of in situ TEM half‐cell setup. Time‐lapse TEM images of Li^+^ insertion in *γ*‐LGVO‐0.09‐NW at b) 10.3 and c) 22.6 s (very obvious strain‐fringe movement can be clearly observed in dashed ellipse). d) SAED pattern of lithiated *γ*‐LGVO‐0.09‐NW. e) Ex situ HRTEM images of pristine (OCV), lithiated (0.2 V), and delithiated (3.0 V) *γ*‐LGVO‐0.09 samples.

Advanced in situ transmission electron microscopy (TEM) technology is employed to investigate the morphology and crystal‐structure evolution of *γ*‐LGVO‐0.09 nanowires (*γ*‐LGVO‐0.09‐NW) during lithiation/delithiation.^[^ ^16^ ^]^ The schematic diagram of the in situ TEM experiment is illustrated in **Figure** **2**a. *γ*‐LGVO‐0.09‐NW is selected for the working electrode since nanoparticles are more suitable for the TEM observation than microparticles (Figure S5, Supporting Information). The strain fringes arising from the Li^+^ insertion into the *γ*‐LGVO‐0.09 nanoparticles exhibit significant movement as highlighted in Figure 2b,c, but the morphology and volume variations of *γ*‐LGVO‐0.09‐NW are negligible (also see Video S1, Supporting Information). The selected area electron diffraction (SAED) pattern of *γ*‐LGVO‐0.09‐NW at the lithiated state (Figure 2d) reveals negligible changes in the lattice spacings of the (101), (210), (011), (111), (211), and (301) planes, verifying the small variations in the lattice constants. In addition, the ex situ high‐resolution TEM (HRTEM) results of *γ*‐LGVO‐0.09 microparticles show that the interlayer spacings of (201) plane at the pristine, lithiated and delithiated states are all 0.374 nm (Figure 2e). All these in situ and ex situ TEM results confirm the intercalation nature of *γ*‐LGVO‐0.09 with small volume change during lithiation/delithiation.

As can be seen in Figure 1b, the wide Li^+^ channels within the *γ*‐LGVO‐0.09 lattice offer spacious sites for the inserted Li^+^ ions, which can decrease the electrostatic repulsion between the neighboring metal ions (V^5+^, V^4+^, V^3+^, Ge^4+^, and Li^+^) and the inserted Li^+^ ions, thus buffering the unit‐cell‐volume change.^[^ ^4e,h^ ^]^ In addition, the volume expansion of the VO_4_ tetrahedra after the reduction of the smaller‐sized V^5+^ ions to the larger‐sized V^4+^ or V^3+^ ions can be accommodated by the surrounding LiO_4_ tetrahedra and LiO_5_ trigonal bipyramids with electrochemical inactivity, further buffering the unit‐cell‐volume change.^[^ ^4e^ ^]^ Thus, the wide Li^+^ channels and inactive polyhedral work together to enable the superior volume‐buffering capability of *γ*‐LGVO‐0.09, and are responsible for its “zero‐strain” characteristic.

**Figure 3:**
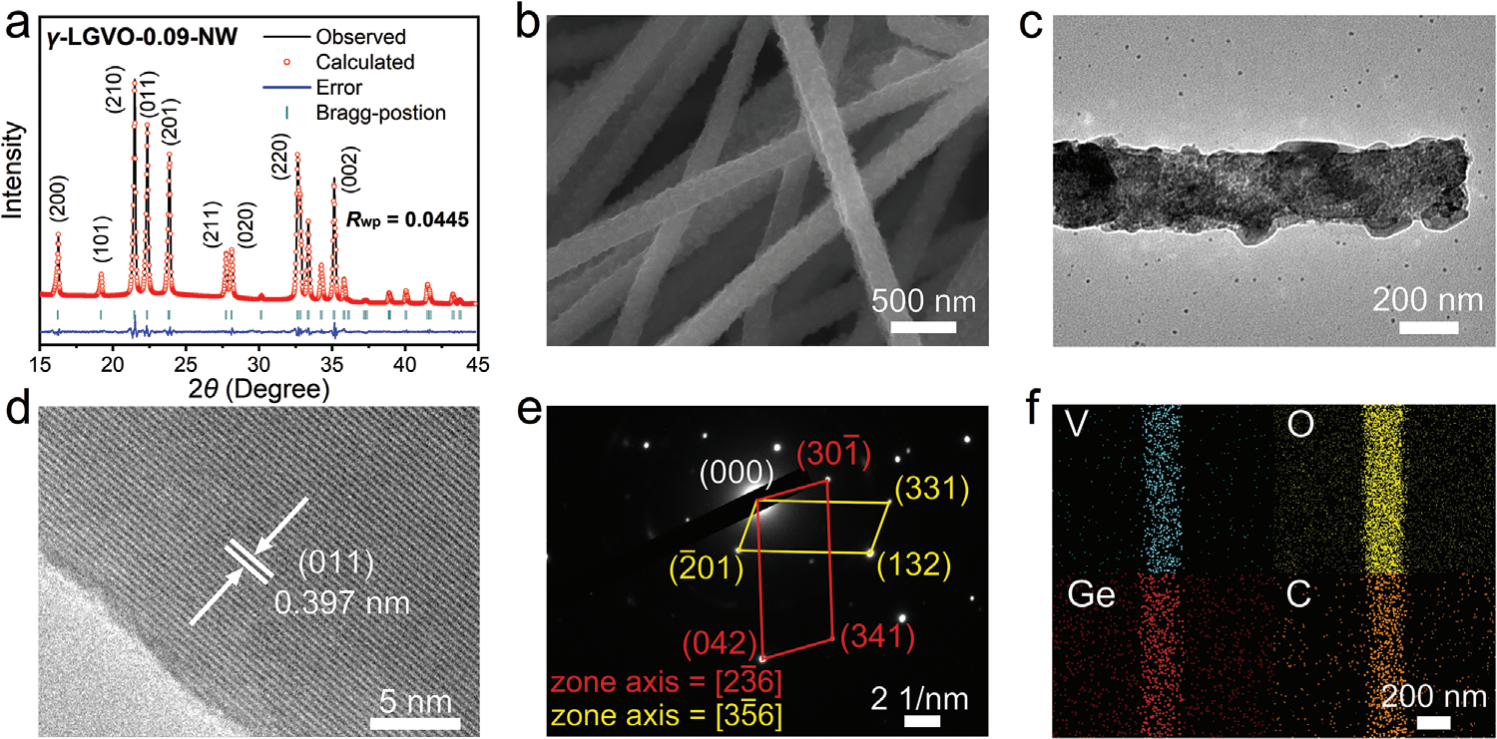
Crystal structure and morphology of *γ*‐LGVO‐0.09‐NW. a) Rietveld‐refined XRD pattern. b) FESEM image. c) TEM image. d) HRTEM image. e) SAED pattern. f) EDS mapping images.

In order to obtain better electrochemical properties (especially the reversible capacity and rate capability, Figures S6 and S7, Supporting Information), nanotechnology is employed to *γ*‐LGVO‐0.09, and pure *γ*‐LGVO‐0.09‐NW with high crystallinity (**Figure** **3**a) is successfully synthesized through electrospinning. *γ*‐LGVO‐0.09‐NW exhibits uniform nanowires with diameters of ≈200 nm and primary‐particle sizes of 15–45 nm (Figure 3b,c), and a large Brunauer–Emmett–Teller (BET) specific surface area of 19.6 m^2^ g^−1^ (Figure S8, Supporting Information). The HRTEM image and SAED pattern confirm the orthorhombic system with *Pnma* space group for *γ*‐LGVO‐0.09‐NW (Figure 3d,e). The energy dispersive spectroscopy (EDS) mapping analyses (Figure 3f) indicate the homogenous Ge, V, O, and C distributions in *γ*‐LGVO‐0.09‐NW, in which the carbon amount is 6.1 wt%, as determined by the thermogravimetry analysis (TGA, Figure S9, Supporting Information).

**Figure 4:**
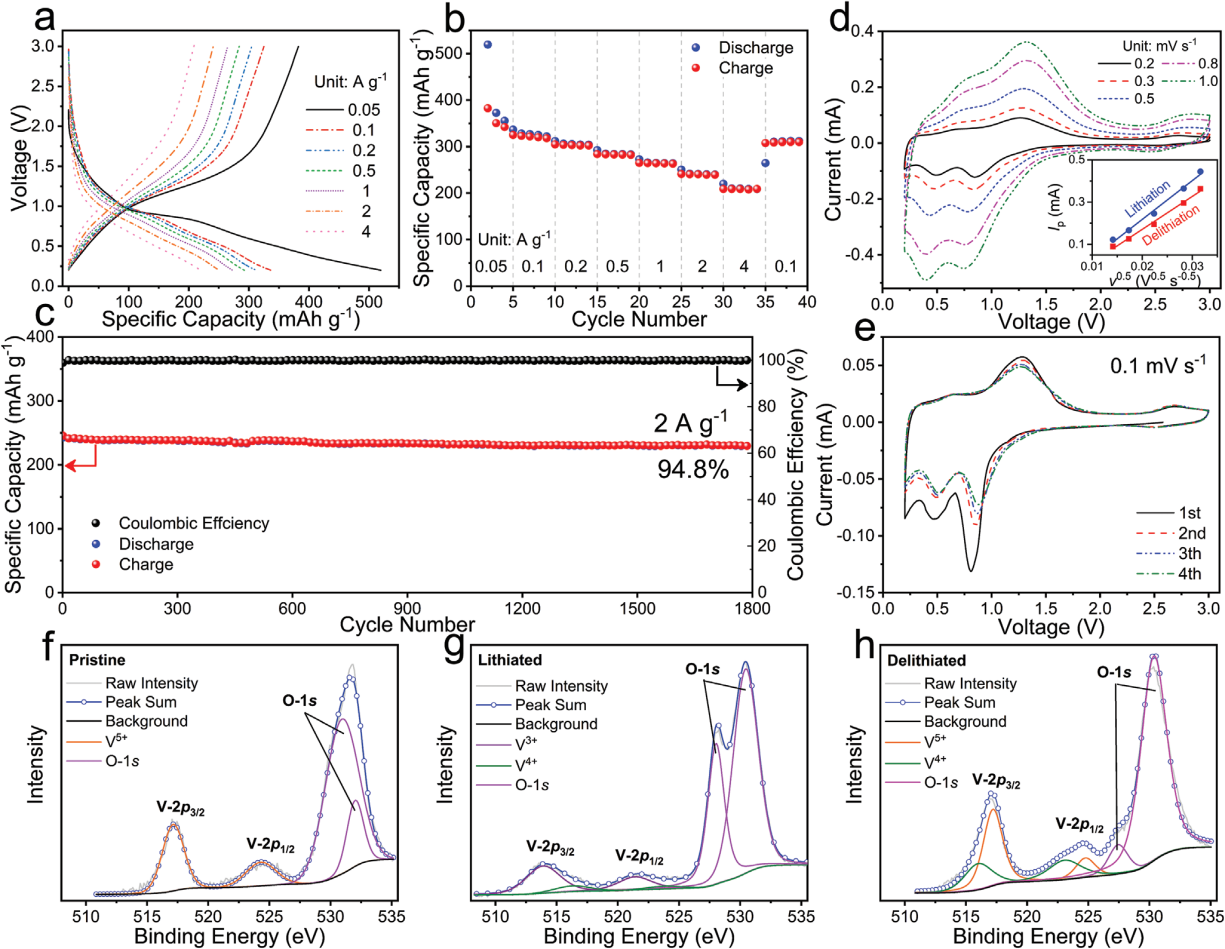
Electrochemical properties, kinetics and redox of *γ*‐LGVO‐0.09‐NW. a) GCD curves for selected cycles at various current densities. b) Rate capability. c) Long‐term cycling stability at 2 A g^−1^ over 1800 cycles (after rate‐capability test). d) CV curves at various scan rates (inset: relationship between peak current (*I*_p_) and square root of scan rate (*v* ^0.5^) for intensive cathodic/anodic peak). e) CV curves for first four cycles at 0.1 mV s^−1^. Ex situ V‐2*p* and O‐1*s* XPS spectra of f) pristine, g) lithiated, and h) delithiated samples.

Figure S10 (Supporting Information) presents the initial three‐cycle galvanostatic charge–discharge (GCD) curves of the *γ*‐LGVO‐0.09‐NW/Li half cell within a potential range of 3.0–0.2 V at a current density of 0.05 A g^−1^. In the initial cycle, *γ*‐LGVO‐0.09‐NW delivers a large reversible capacity of 383 mAh g^−1^ with a proper working potential of 1.03 V. Its reversible capacity is stabilized at 346 mAh g^−1^ in the subsequent cycles, surpassing those of most intercalation‐type anode materials previously reported (Table S4, Supporting Information). When the current density increases to 0.1, 0.2, 0.5, 1, 2, and 4 A g^−1^, it retains large average reversible capacities of 320, 303, 283, 264, 240, and 209 mAh g^−1^, respectively (**Figure** **4**a,b). When the current density returns 0.1 A g^−1^, its average reversible capacity recovers 310 mAh g^−1^, indicating its good electrochemical stability. In addition, its superior long‐term cycling stability is identified through cycling 1800 cycles at 2 A g^−1^ with high capacity‐retention percentage of 94.8% (Figure 4c), which is better than that of *γ*‐LGVO‐0.06‐NW with larger maximum unit‐cell‐volume change (Figure S10, Supporting Information), among the best results in the research field of intercalating anode materials (Table S4, Supporting Information), and undoubtedly due to its “zero‐strain” characteristic. The above comprehensively good electrochemical properties fully demonstrate that “zero‐strain” *γ*‐LGVO‐0.09‐NW is a very promising and practical anode material for high‐energy, high‐power, safe, and stable LIBs.

From the cyclic voltammetry (CV) data of *γ*‐LGVO‐0.09‐NW recorded at different scan rates (Figure 4d), its apparent Li^+^ diffusion coefficients (*D*_Li_) are determined (Figure 4d inset) using the Randles–Sevcik equation (details in the Supporting Information).^[^ ^17^ ^]^ During lithiation and delithiation, its *D*_Li_ values, respectively, reach 4.5 × 10^−12^ and 2.9 × 10^−12^ cm^2^ s^−1^, which match well with the galvanostatic intermittent titration technique (GITT) results (Figure S11, Supporting Information), and larger than those of other Li_3_VO_4_‐based anode materials and most intercalating anode materials (Table S5, Supporting Information). The large Li^+^ diffusion coefficients of *γ*‐LGVO‐0.09‐NW are partially attributed to its open crystal structure with wide Li^+^ transport channels. In addition, its enhanced electronic conductivity (1.0 × 10^−7^ S cm^−1^ for light yellow *γ*‐LGVO‐0.09 vs 5.9 × 10^−9^ S cm^−1^ for white *β*‐Li_3_VO_4_, Figure S12, Supporting Information) and carbon compositing, respectively, enable faster electron conduction within and between its primary particles, and its nanowire morphology enables shorter Li^+^ transport distances and a larger electrochemical‐reaction area, which also contribute to its large apparent Li^+^ diffusion coefficients.^[^ ^18^ ^]^ All these merits work together, leading to its high rate capability.

From the initial three‐cycle CV curves of *γ*‐LGVO‐0.09‐NW (Figure 4e), it is found that the initial cathodic branch is different from the subsequent overlapped cycles,^[^ ^19^ ^]^ which is in good agreement with the GCD curves (Figure S13, Supporting Information). Two cathodic peaks at 0.81 and 0.47 V combined with two anodic peaks at 1.29 and 0.64 V can correspond to the reversible reduction/oxidation based on the V^5+^/V^4+^ and V^4+^/V^3+^ redox couples.^[^ ^11^, ^20^ ^]^ The asymmetry of the CV curves, which is commonly seen in Li^+^‐storage materials, may be due to the different electrochemical kinetics between lithiation and delithiation (note that the Li^+^ diffusion coefficients during lithiation and delithiation are different, Figure 4d). To identify the two‐electron transfer per vanadium ion, ex situ X‐ray photoelectron spectroscope (XPS) characterizations of the *γ*‐LGVO‐0.09‐NW electrodes at different discharge/charge states are performed within 3.0–0.2 V (Figure 4f,g,h; and Figure S14, Supporting Information). The V‐2*p* spectrum at the pristine (OCV) state includes a V‐2*p*_1/2_ and V‐2*p*_3/2_ doublet at 524.3 and 517.2 eV (Figure 4f), indicating that the vanadium element exists as V^5+^ (100%), as expected. When discharge to 0.2 V (Figure 4g), the lower doublet at 523.5 and 516.3 eV can be assigned to V^4+^ (13%), and the further lower doublet at 521.2 and 514.0 eV can be assigned to V^3+^ (87%).^[^ ^21^ ^]^ When charge to 3.0 V (Figure 4h), the chemical state of vanadium significantly recovers, obtaining a combination of V^5+^ (68%) and V^4+^ (32%). The fact that a small portion of V^4+^ ions are not oxidated to V^5+^ is reasonable since the inserted Li^+^ ions cannot be totally extracted during initial charge.^[^ ^19^ ^]^

**Figure 5:**
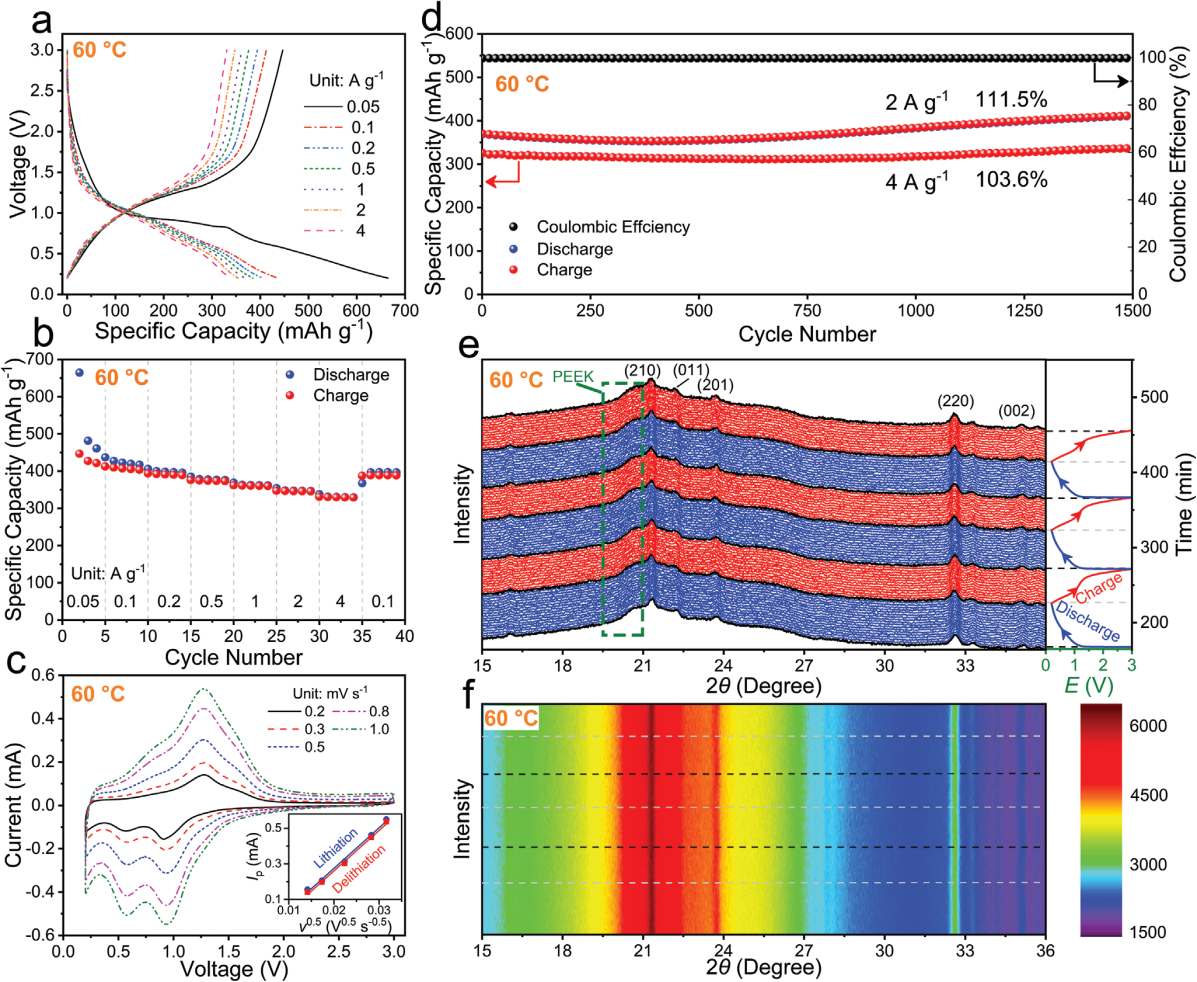
Electrochemical properties and crystal‐structure evolution of *γ*‐LGVO‐0.09‐NW at 60 ℃. a) GCD curves for selected cycles at various current densities. b) Rate capability. c) CV curves at various scan rates (inset: relationship between *I*_p_ and *v* ^0.5^ for intensive cathodic/anodic peak). d) Long‐term cycling stability at 2 and 4 A g^−1^ over 1500 cycles (after rate‐capability test). e) Pristine and f) 2D in situ XRD patterns with corresponding GCD curves after initial cycle within 3.0–0.2 V at 0.5 A g^−1^. PEEK: polyetheretherketone.

At the high temperature of 60 ℃, *γ*‐LGVO‐0.09‐NW offers a very large reversible capacity of 425 mAh g^−1^ with an average working potential of 1.02 V at 0.05 A g^−1^ in the initial cycle (**Figure** **5**a; and Figure S15, Supporting Information), which is 42 mAh g^−1^ larger than that at 25 ℃. This excess Li^+^ intercalation is undoubtedly promoted by the high temperature.^[^ ^7b^ ^]^ At 0.1, 0.2, 0.5, 1, 2, and 4 A g^−1^, its reversible capacity at 60 ℃ retains 407, 398, 378, 363, 347, and 331 mAh g^−1^, respectively (Figure 5a,b). Attractively, its 4 vs 0.1 A g^−1^ capacity‐retention percentage at 60 ℃ reaches 80.2%, obviously larger than that at 25 ℃ (64.2%), which can be ascribed to its faster Li^+^ diffusivity at 60 ℃ with larger *D*_Li_ values of 6.9 × 10^−12^ and 6.7 × 10^−12^ cm^2^ s^−1^, respectively, for lithiation and delithiation (Figure 5c). Besides its large reversible capacities at various current densities, it further shows excellent long‐term (1500 cycles) cycling stability with capacity‐retention percentages of 111.5% and 103.7%, respectively, at 2 and 4 A g^−1^ (Figure 5d). The capacity‐increases during the different cycling can be ascribed to the easy *γ*‐LGVO‐0.09‐NW activation at the high temperature.^[^ ^1^, ^22^ ^]^ In stark contrast, the capacity‐retention percentage for anatase TiO_2_ nanocrystals is only ≈20% at 100 mA g^−1^ over 50 cycles,^[^ ^7b^ ^]^ and that for porous Li_4_Ti_5_O_12_ microspheres is only 20.9% at 5C over 500 cycles at the same high temperature;^[^ ^23^ ^]^ and that for carbon‐coated LiFePO_4_ nanoparticles is only ≈40% at 5C over 300 cycles at 55 ℃.^[^ ^24^ ^]^ Therefore, *γ*‐LGVO‐0.09‐NW is superior anode material particularly suitable for high‐temperature applications.

The evolution of the in situ XRD patterns recorded from the *γ*‐LGVO‐0.09/Li in situ cell at 60 ℃ (Figure 5e,f) is very similar to that at 25 ℃ (Figure 1c,d). The characteristic peaks of *γ*‐LGVO‐0.09 also show negligible shifts. The resultant unit‐cell‐volume change is slightly increased to 0.107%, which is undoubtedly due to the larger reversible Li^+^‐storage capacity (i.e., more Li^+^ ions intercalated) at 60 ℃. This only slight increase fully demonstrates the superior volume‐buffering capability of the *γ*‐LGVO‐0.09 compound at not only the room temperature but also the high temperature, and well explains the excellent high‐temperature cycling stability of *γ*‐LGVO‐0.09‐NW. Our work demonstrates that the “zero‐strain” characteristic of an electrode material can greatly benefit its high‐temperature cycling stability.

## 3 Conclusion

Selective doping is explored as a general strategy to tailor the maximum unit‐cell‐volume change of an intercalating Li^+^‐storage material, and the typical case of Ge^4+^/Si^4+^ doped *γ*‐Li_3_VO_4_ is demonstrated. The tailoring follows the law of doping with large‐sized ions → increasing unit‐cell volume (i.e., increasing crystal‐structure openness) → decreasing maximum unit‐cell‐volume change. *γ*‐Li_3.09_Ge_0.09_V_0.91_O_4_ with 9% Ge^4+^ doping shows an extremely small maximum unit‐cell‐volume change of 0.016%, which is the smallest among all the known Li^+^‐storage materials. As a result, *γ*‐Li_3.09_Ge_0.09_V_0.91_O_4_ nanowires exhibit excellent cycling stability (capacity‐retention percentage of 94.8% after 1800 cycles at 2 A g^−1^). This material further delivers a large reversible capacity (383 mAh g^−1^ at 0.05 A g^−1^), proper working potential (1.03 V) and high rate capability (4 vs 0.1 A g^−1^ capacity‐retention percentage of 64.2%). Attractively, at the high temperature of 60 °C, it retains excellent cycling stability (capacity‐retention percentages of 111.5% and 103.7% after 1500 cycles at 2 and 4 A g^−1^, respectively), and enhances the reversible capability (425 mAh g^−1^ at 0.05 A g^−1^) and rate capability (4 vs 0.1 A g^−1^ capacity‐retention percentage of 80.2%), demonstrating the superiority of “zero‐strain” materials during high‐temperature operation. The insight gained in this work can provide a guide for the crystal‐structure and composition design of highly‐stable Li^+^‐storage materials at room and high temperatures.

## 4 Experimental Section

### Material Preparations

According to the formula of Li_3+_ *_x_* Ge*_x_* V_1−_ *_x_* O_4_ (*x* = 0.09), *γ*‐phase Li_3.09_Ge_0.09_V_0.91_O_4_ (*γ*‐LGVO‐0.09) microparticles were prepared by calcinating a mixture composed of 15.68 mmol Li_2_CO_3_ (99.5%, Macklin, 1% lithium excess), 0.90 mmol GeO_2_ (99.99%, Macklin), and 9.19 mmol NH_4_VO_3_ (99%, Macklin) at 750 °C for 3 h and at 800 °C for another 15 h (heating rate of 10 °C min^−1^), which was ball‐milled for 1 h in a high‐energy ball‐milling machine (SPEX 8000M) before the calcination. For comparison, other Ge^4+^ doped Li_3_VO_4_ (*γ*‐LGVO‐*x*, *x* = 0.06, 0.08, and 0.10) microparticles were prepared by the same method using a Li_2_CO_3_ : GeO_2_ : NH_4_VO_3_ molar ratio of 1.01(3+*x*) : 2*x* : 2(1−*x*), and Si^4+^ doped Li_3_VO_4_ (*γ*‐LSVO‐*x*, *x* = 0.06, 0.08, and 0.10) microparticles were prepared by the same method using a Li_2_CO_3_ : SiO_2_ (99.99%, Macklin): NH_4_VO_3_ molar ratio of 1.01(3+*x*) : 2*x* : 2(1−*x*).

*γ*‐phase Li_3.09_Ge_0.09_V_0.91_O_4_ nanowires (*γ*‐LGVO‐0.09‐NW) were synthesized by electrospinning and postcalcination. 32.48 mmol LiOH (98%, Macklin) with 3% lithium excess, 0.90 mmol GeO_2_ (99.99%, Macklin), and 0.5 g citric acid (99.5%, Macklin) were dissolved in 10 mL deionized water, stirring until a transparent solution was formed. Thereafter, 9.19 mmol NH_4_VO_3_ (99%, Macklin), 0.6 g ascorbic acid (99.99%, Macklin), 0.5 mL acetic acid (99.5%, Macklin), and 0.9 g polyvinylpyrrolidone (*M*_w_ = 1 300 000, Macklin) were successively added to the solution, stirring for 5 h to obtain the electrospinning solution. The electrospinning process was operated at a 21 kV applying voltage, 0.5 mL h^−1^ pumping rate and 15 cm working distance. The collected as‐spun nanowires were heated at 380 °C for 10 h in air to stabilize the nanowire structure and further calcinated at 750 °C for 15 h in argon (heating rate of 2 °C min^−1^), finally achieving *γ*‐LGVO‐0.09‐NW.

### Material Characterizations

The XRD patterns of all the samples were collected on a commercial X‐ray diffractometer (Rigaku Ultima IV) with a step scanning rate of 10° min^−1^ from 10° to 70° (2*θ*). The lattice constants were determined by Rietveld refinements conducted on the General Structure Analysis System (GSAS).^[^ ^8^ ^]^ The morphologies, microstructures, element compositions, and SEAD patterns were examined on a field emission scanning electron microscope (FESEM, JEOL JSM‐7800F) equipped with an EDS (OXFORD X‐Max), and a HRTEM (JEOL JEM‐2100Plus). The measurements of the BET specific surface areas were performed on a nitrogen adsorption–desorption device (Autosorb iQ3, Quantachrome). The mass percentage of carbon in *γ*‐LGVO‐0.09‐NW was determined using a thermogravimetry analyzer (TGA, TGA 2, Mettler Toledo). The chemical states of ions in *γ*‐LGVO‐0.09‐NW at different discharge/charge states were recorded on an XPS (PHI5000 Versaprobe III). The electronic‐conductivity test of *γ*‐LGVO‐0.09/*β*‐Li_3_VO_4_ was performed using a two‐probe direct‐current method based on a compact *γ*‐LGVO‐0.09/*β*‐Li_3_VO_4_ pellet with a diameter of 1 cm and a thickness of 0.15 cm, which was prepared by pressing the *γ*‐LGVO‐0.09/*β*‐Li_3_VO_4_ microparticles within a specially‐designed module.

### Electrochemical Measurements

The electrochemical properties were examined using CR2032‐type coin cells assembled in a glove box filled with argon. The electrolyte was 1 m LiPF_6_ dissolved in mixture of ethylene carbonate, diethylene carbonate, and dimethyl carbonate (1 : 1 : 1 in volume, LB‐4740‐034, Jiangsu Guotaihuarong). Microporous polypropylene films (Celgard 2325) served as separators, and lithium metal foils served as counter/reference electrodes. The working electrodes were composed of active materials (*γ*‐LGVO‐0.09‐NW or *γ*‐LGVO‐0.09, 1.5 mg cm^−2^), polyvinylidene fluoride, and conductive carbon (Timcal Super C65) in a mass ratio of 7 : 1 : 2 on copper‐foil current collectors and vacuum‐dried at 110 °C for 12 h. The electrochemical properties were tested using GCD and GITT on a multichannel battery tester (CT‐3008, Shenzhen Neware). Short current pulses at 50 mA g^−1^ for 10 min and long rest intervals for 3 h were used for the GITT tests. The cyclic voltammetry (CV) studies were carried out on an electrochemical workstation (CHI660E, Shanghai Chenhua). The temperatures of the above electrochemical measurements were controlled at 25 and 60 °C in a temperature‐variable oven (SPM‐258, Ningbo Xinjiangnan).

### In Situ Characterizations

For the in situ XRD tests, the working electrode was prepared by the same way with that in the coin cells except that a beryllium foil was employed as the current collector. The in situ cell was assembled in a specially‐designed module (LIB‐LHTXRD‐LN, Beijing Scistar Technology),^[^ ^13^ ^]^ in which the beryllium foil further served as the X‐ray transmission window. The in situ XRD test at 60 °C was performed with a homemade temperature‐control device, and a polyetheretherketone (KEEK) dome was coated on the module for accurately controlling the temperature.^[^ ^25^ ^]^ The in situ XRD patterns were collected in a 2*θ* range from 15° to 36° within 3.0–0.2 V at a current density of 0.5 A g^−1^. In situ TEM performed on an electrochemical holder from the X‐mech Center (Zhejiang University) was employed to observe the Li^+^ transmission within *γ*‐LGVO‐0.09‐NW during lithiation.^[^ ^16^ ^]^ The working electrode was *γ*‐LGVO‐0.09‐NW on a silver rod. The counter electrode was lithium metal on a tungsten rod. The lithium metal was naturally coated with Li_2_O to form a solid electrolyte layer. An external voltage was applied to drive lithium metal → Li_2_O → *γ*‐LGVO‐0.09‐NW lithiation when the two electrodes were in contact.

## Conflict of Interest

The authors declare no conflict of interest.

## Supporting information

Supporting Information

Supplemental Video 1

## Data Availability

Research data are not shared.
