# Supplementary material for: Selective Doping to Controllably Tailor Maximum Unit‐Cell‐Volume Change of Intercalating Li+‐Storage Materials: A Case Study of γ Phase Li3VO4
Source: Adv Sci (Weinh). 2022 Jun 24;9(24):2106003. doi: 10.1002/advs.202106003 (PMC9405516; doi:10.1002/advs.202106003)
Supplement: Supplementary file 1 — Supporting Information [file ADVS-9-2106003-s001.pdf]

ADVANCED SCIENCE
Open Access

Supporting Information

for *Adv. Sci.*, DOI 10.1002/advs.202106003

Selective Doping to Controllably Tailor Maximum Unit-Cell-Volume Change of Intercalating Li^{+}-Storage Materials: A Case Study of γ Phase Li_3VO_4

*Jianbin Deng, Changpeng Lv, Tian Jiang, Siyuan Ma, Xuehua Liu and Chunfu Lin**

Supporting Information

Selective Doping to Controllably Tailor Maximum Unit-Cell-Volume Change of Intercalating Li^+-Storage Materials: A Case Study of *γ* Phase Li_3VO_4

*Jianbin Deng, Changpeng Lv, Tian Jiang, Siyuan Ma, Xuehua Liu, and Chunfu Lin**

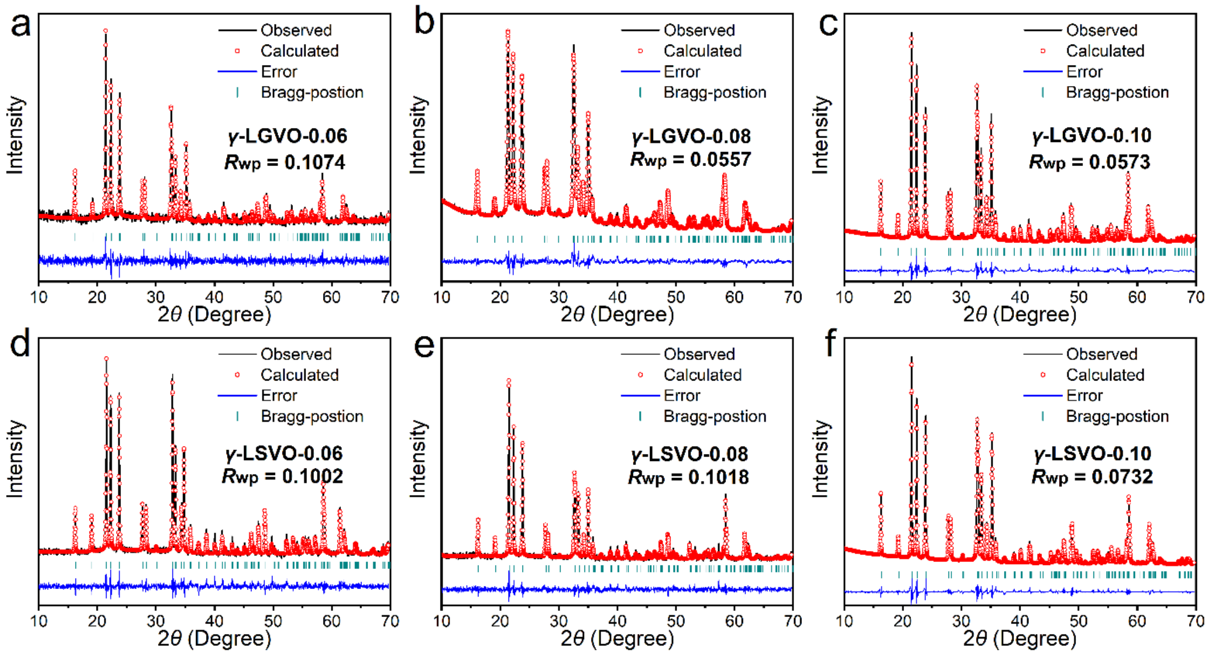

Figure S1. XRD patterns of a) *γ*-LGVO-0.06, b) *γ*-LGVO-0.08, c) *γ*-LGVO-0.10, d) *γ*-LSVO-0.06, e) *γ*-LSVO-0.08 and f) *γ*-LSVO-0.10 with Rietveld refinements.

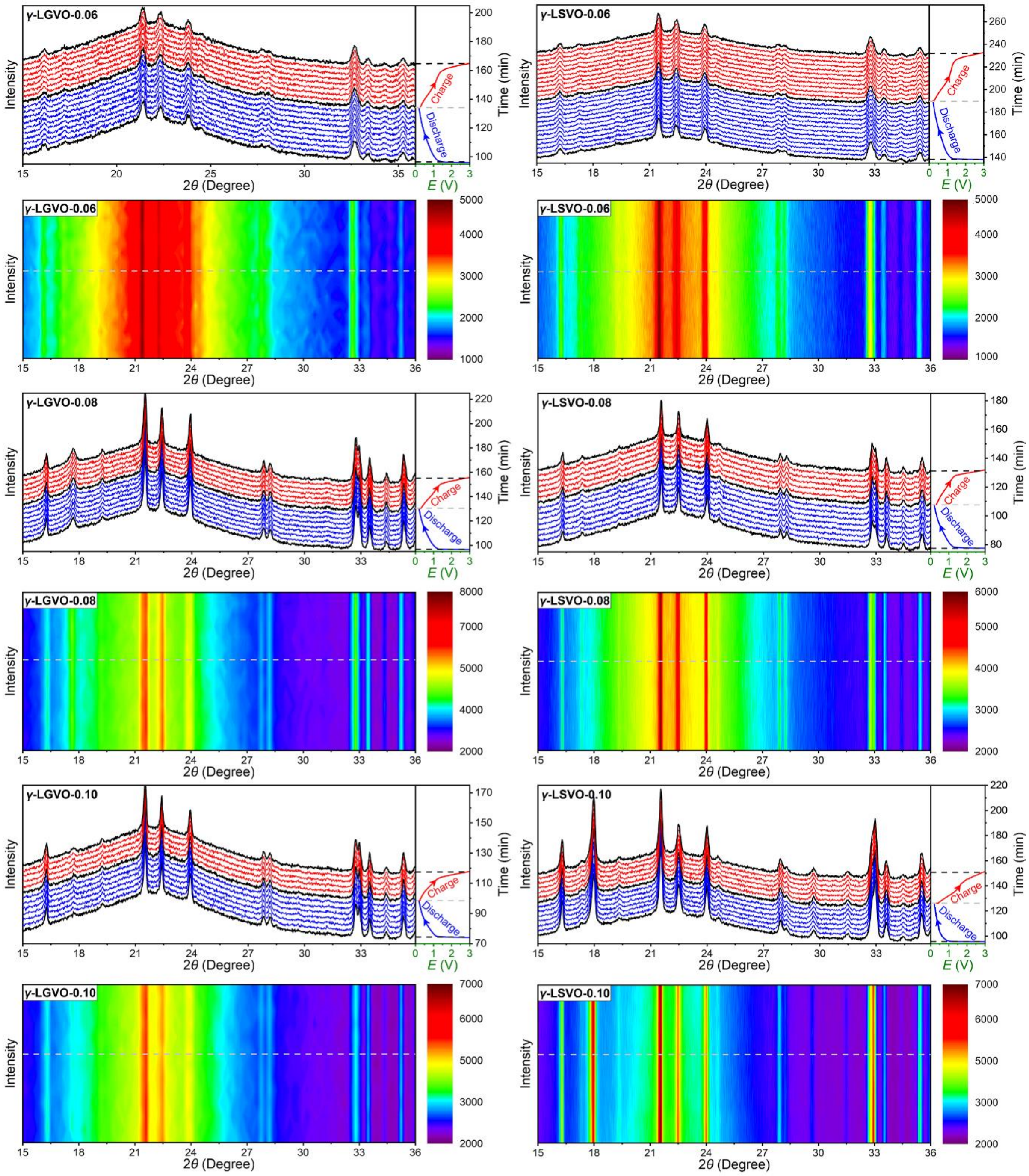

Figure S2. Pristine and two-dimensional *in-situ* XRD patterns of γ-LGVO-*x*, γ-LSVO-*x* (*x* = 0.06, 0.08 and 0.10) with corresponding GCD curves after initial cycle within 3.0–0.2 V at 0.5 A g^{-1}.

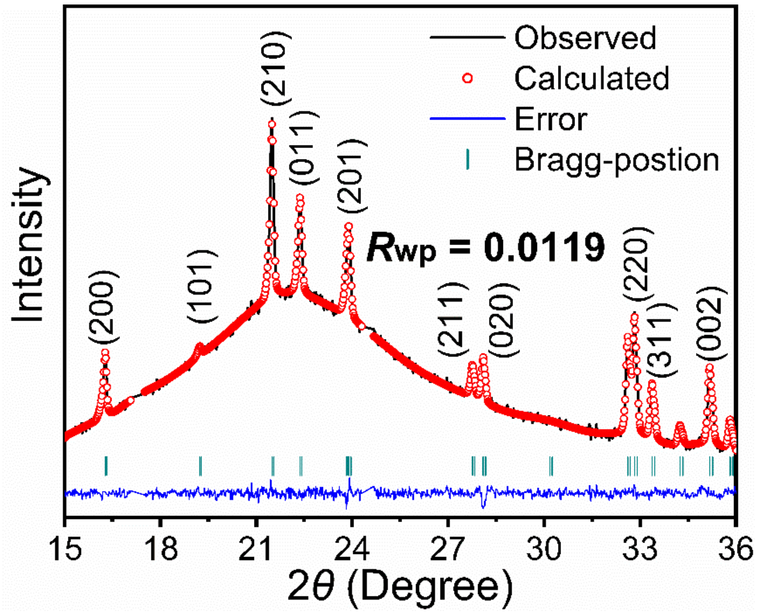

Figure S3. Rietveld refinement of initial *in-situ* XRD pattern in Figure 1c.

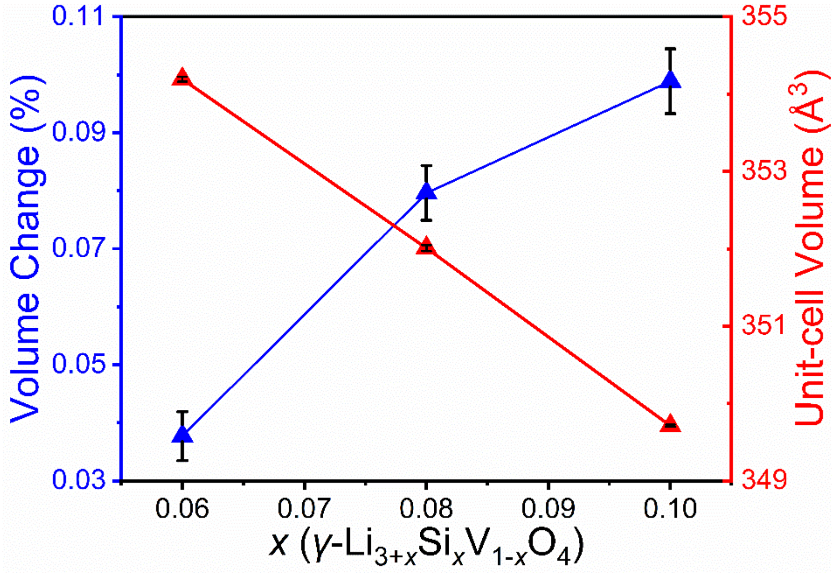

Figure S4. Maximum unit-cell-volume-change percentages and unit-cell volumes of γ-LSVO-x (x = 0.06, 0.08 and 0.10) samples. Error bar represents one standard deviation of uncertainty.

Detailed description of additional examples on doping effects on unit-cell-volume changes of Li^+-storage materials

Additional Example 1: Zr^{4+}–Si^{4+} co-doped $LiFePO_4$

This Zr^{4+}–Si^{4+} co-doping can be expressed as $ZrO_2 + 2SiO_2 = Zr_{Fe}^{\cdot\cdot} + 2Si_P' + 6O_O^{\times}$. Fe^{2+} and Zr^{4+} respectively have ion sizes of 0.78 and 0.72 Å in the octahedral coordination, and P^{5+} and Si^{4+} respectively have ion sizes of 0.17 and 0.26 Å in the tetrahedral coordination [S1]. Since 0.72+0.26×2>0.78+0.17×2, the unit-cell volume of $Li(Fe_{0.875}Zr_{0.125})(P_{0.75}Si_{0.25})O_4$ is increased, leading to its smaller maximum unit-cell-volume change and thus better cycling stability [S2].

Additional Example 2: Cr^{3+}/Ti^{3+} substituted $Li_4Ti_5O_{12}$

$LiCrTiO_4$ can be regarded as Cr^{3+} substituted spinel $Li_4Ti_5O_{12}$ with $3Cr_2O_3 = 4Cr_{Ti}' + 2Cr_{Li}^{\cdot\cdot} + 9O_O^{\times}$. Ti^{4+}, Li^+ and Cr^{3+} respectively have ion sizes of 0.605, 0.76 and 0.615 Å in the octahedral coordination [S1]. Since 0.615×6<0.605×4+0.76×2, the unit-cell volume of $LiCrTiO_4$ is decreased, leading to its larger maximum unit-cell-volume variation (0.7% for $LiCrTiO_4$ *vs.* −0.2% for $Li_4Ti_5O_{12}$ after lithiation) [S3,S4].

$LiTi_2O_4$ can be regarded as Ti^{3+} substituted $Li_4Ti_5O_{12}$. Ti^{3+} has an ion size of 0.67 Å in the octahedral coordination [S1]. Since 0.67×6>0.605×4+0.76×2, the unit-cell volume of $LiTi_2O_4$ is increased, leading to its smaller maximum unit-cell-volume variation (−1.0% for $LiCrTiO_4$ *vs.* −0.2% for $Li_4Ti_5O_{12}$ after lithiation) [S4,S5].

Additional Example 3: Ni^{2+}–Nb^{5+} co-substituted $GaNb_{11}O_{29}$

$Ni_2Nb_{34}O_{87}$ can be regarded as Ni^{2+}–Nb^{5+} co-substituted orthohombic $GaNb_{11}O_{29}$ with $4NiO + Nb_2O_5 = 4Ni_{Ga}' + 2Nb_{Ga}^{\cdot\cdot} + 9O_O^{\times}$. Ga^{3+}, Ni^{2+} and Nb^{5+} respectively have ion sizes of 0.62, 0.69 and 0.68 Å in the octahedral coordination [S1]. Since 0.69×4+0.64×2>0.62×6, the

unit-cell volume of $Ni_2Nb_{34}O_{87}$ is increased (2277 Å^3 for $Ni_2Nb_{34}O_{87}$ *vs.* 2244 Å^3 for $GaNb_{11}O_{29}$), leading to its smaller maximum unit-cell-volume change (6.71% for $Ni_2Nb_{34}O_{87}$ *vs.* 7.15% for $GaNb_{11}O_{29}$) and better cycling stability (101.7% capacity retention at 10C over 1500 cycles for $Ni_2Nb_{34}O_{87}$ *vs.* 66.9% capacity retention at 10C over 1000 cycles for $GaNb_{11}O_{29}$) [S6,S7].

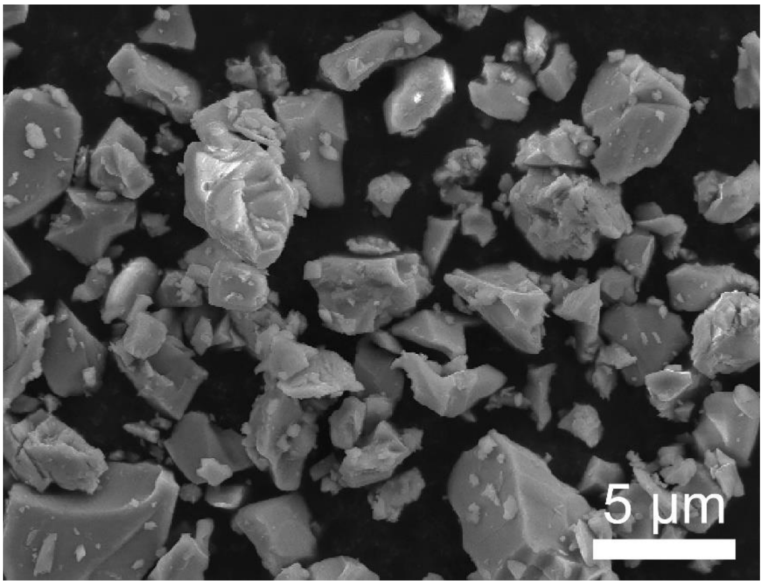

Figure S5. FESEM image of γ-LGVO-0.09.

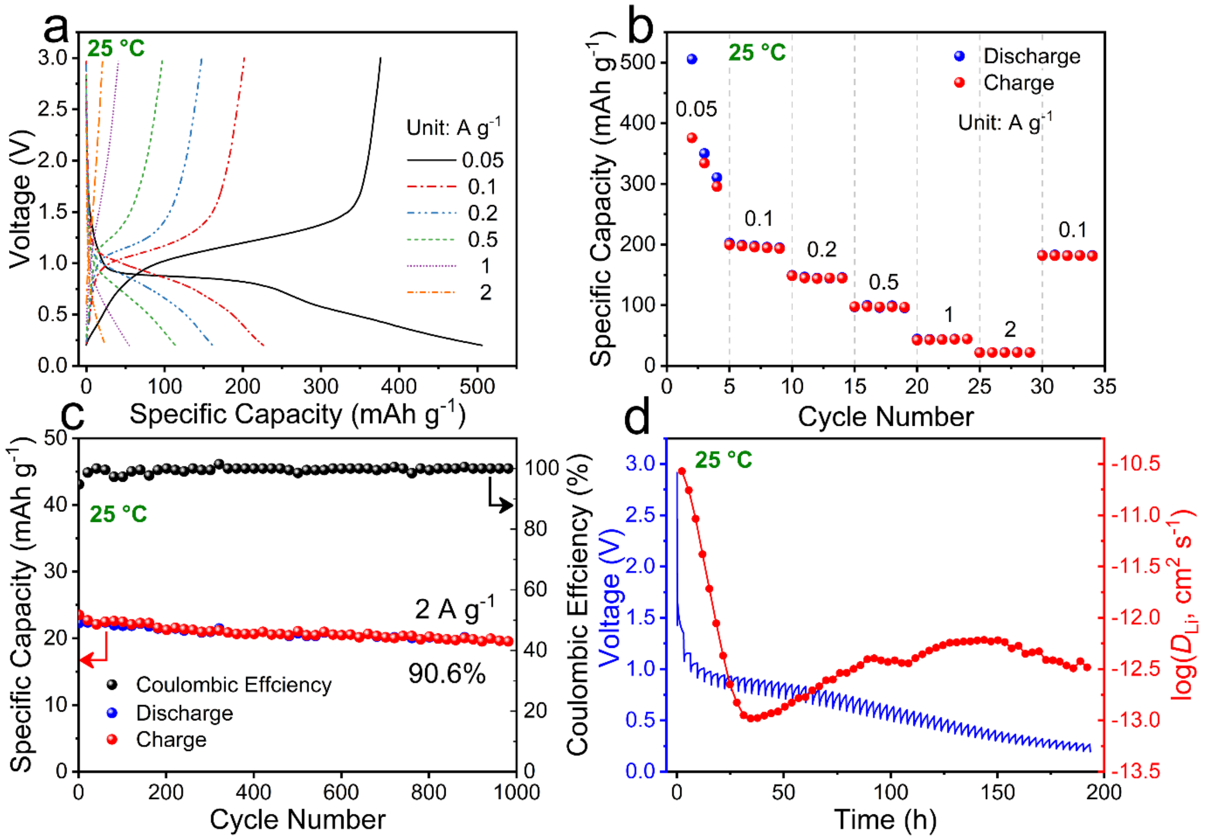

Figure S6. Electrochemical properties of *γ*-LGVO-0.09 at 25 °C. a) GCD curves for selected cycles at various current densities. b) Rate capability. (c) Long-term cycling stability at 2 A g^{-1} over 1000 cycles (after rate-capability test). (d) Second-cycle GITT lithiation curve and D_{Li} variation. The higher rate capability of *γ*-LGVO-0.09-NW than that of *γ*-LGVO-0.09 can be due to the larger Li^+ diffusion coefficients, significantly smaller primary-particle sizes, and additional carbon compositing for *γ*-LGVO-0.09-NW.

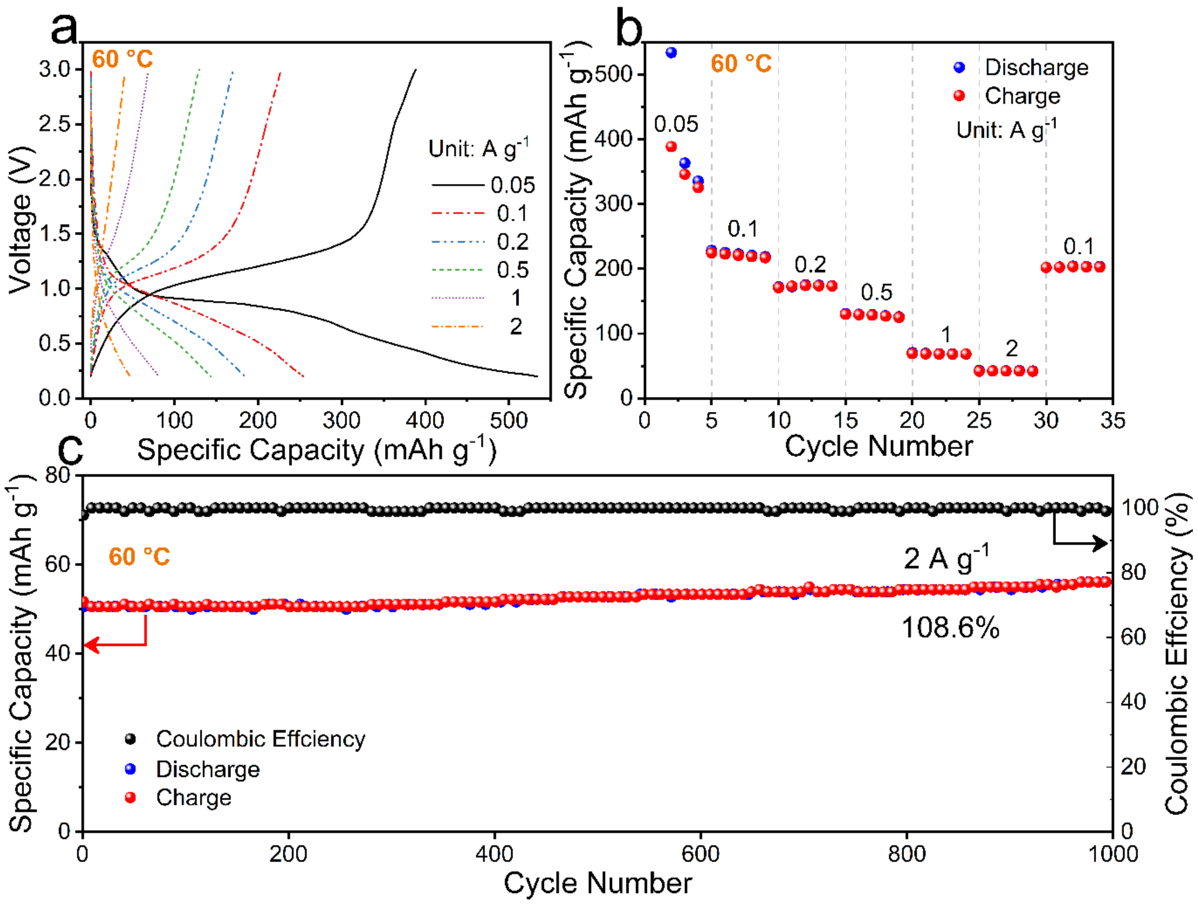

Figure S7. Electrochemical properties of γ-LGVO-0.09 at 60 °C. a) GCD curves for selected cycles at various current densities. b) Rate capability. (c) Long-term cycling stability at 2 A g^{-1} over 1000 cycles (after rate-capability test).

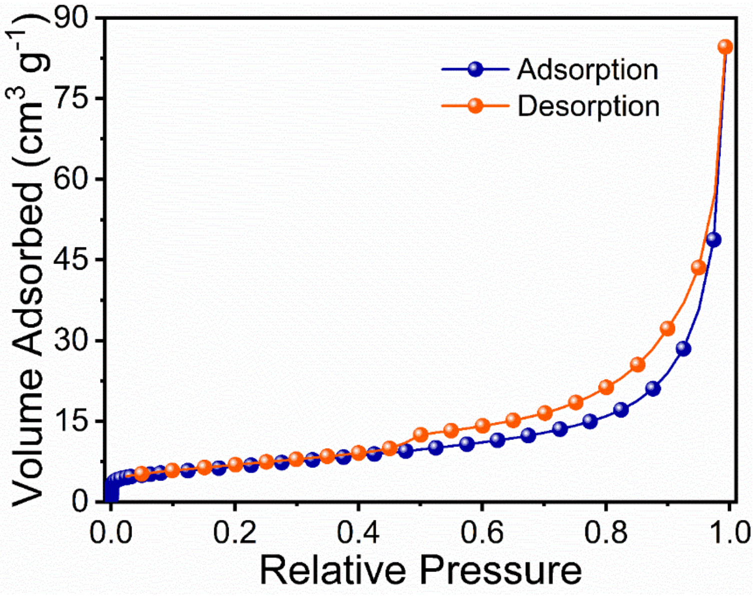

Figure S8. Nitrogen adsorption–desorption isotherm of *γ*-LGVO-0.09-NW.

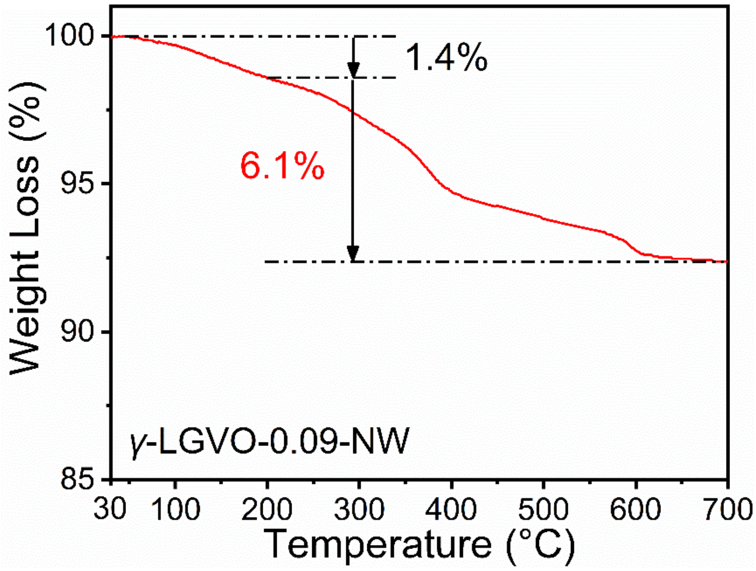

Figure S9. TGA curve of *γ*-LGVO-0.09-NW.

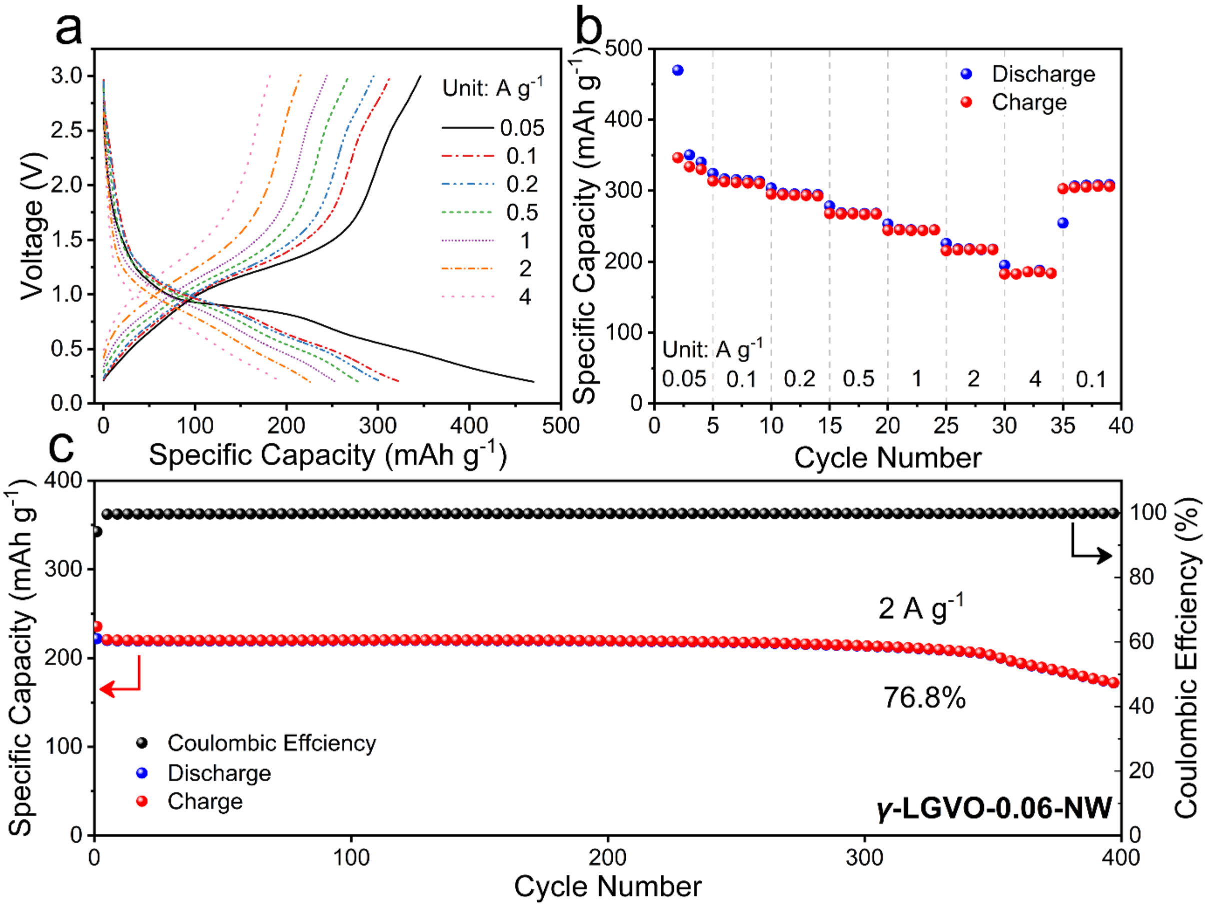

Figure S10. Electrochemical properties of γ-LGVO-0.06-NW at 25 °C. a) GCD curves for selected cycles at various current densities. b) Rate capability. (c) Long-term cycling stability at 2 A g^{-1} over 400 cycles (after rate-capability test). Compared with γ-LGVO-0.09-NW, γ-LGVO-0.06-NW shows similar capacities and rate capability, but poorer cycling stability. γ-LGVO-0.06 is selected for the comparison since the lower limit of the x value for γ-$Li_{3+x}Ge_xV_{1-x}O_4$ is 0.06 (*i.e.*, 6 at.% Ge^{4+} doping).

Calculations of apparent Li^+ diffusion coefficients through CV

The apparent Li^+ diffusion coefficients of *γ*-LGVO-0.09-NW at 25 and 60 °C are determined from its CV data at different scan rates (**Figure 4d,5c**). It is found that the peak current of the intensive cathodic/anodic reaction I_p is in proportional to the square root of the scan rate $v^{0.5}$ (**Figure 4d/Figure 5c** *inset*), which shows the linear semi-infinite diffusion in cathodic and anodic processes. Consequently, the Randles–Sevcik equation (**Equation S1**) can be applied [S8,S9], based on which the Li^+ diffusion coefficient D_{Li} can be calculated.

$$I_p = 0.4463 n^{1.5} F^{1.5} C S R^{-0.5} T^{-0.5} D_{Li}{}^{0.5} \qquad \text{(S1)}$$

where *n*, *F*, *C*, *S*, *R* and *T* are the charge transfer number, Faraday's constant, molar concentration of Li^+ in solid, electrode area, molar gas constant and absolute temperature, respectively. During lithiation and delithiation, the D_{Li} values of *γ*-LGVO-0.09-NW respectively reach 4.5×10^{-12} and 2.9×10^{-12} $cm^2\ s^{-1}$ at 25 °C, and increase to 6.9×10^{-12} and 6.7×10^{-12} $cm^2\ s^{-1}$ at 60 °C.

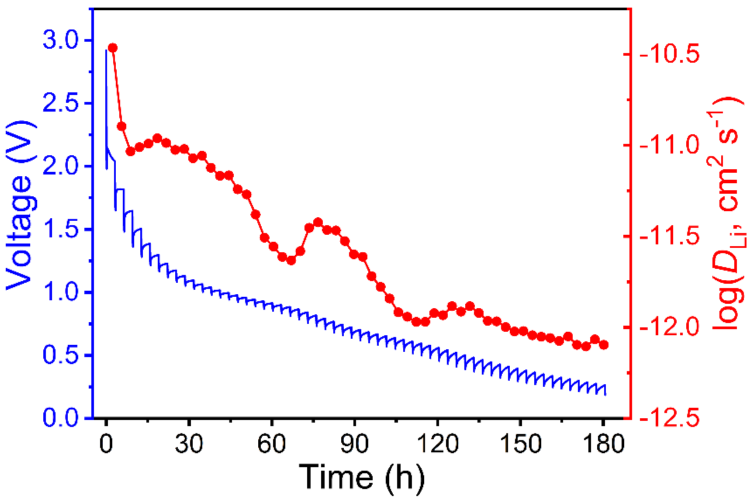

Figure S11. Second-cycle GITT lithiation curve of *γ*-LGVO-0.09-NW and its D_{Li} variation.

Calculations of apparent Li^+ diffusion coefficients through GITT

The apparent Li^+ diffusion coefficients of γ-LGVO-0.09-NW at 25 °C are further determined from its second-cycle GITT lithiation curve (**Figure S11**). The calculation is based on **Equation S2** [S8,S9].

$$D_{\mathrm{Li}} = \frac{4}{\pi\tau}\left(\frac{m_{\mathrm{b}}V_{\mathrm{m}}}{M_{\mathrm{b}}S}\right)^2\left(\frac{\Delta E_{\mathrm{S}}}{\Delta E_{\tau}}\right)^2 \quad (t \ll L^2 \,/\, D_{\mathrm{Li}}) \qquad \text{(S2)}$$

where m_b, V_m, M_b, S, τ and L are respectively the real mass of γ-LGVO-0.09-NW, molar volume of γ-LGVO-0.09-NW, molar mass of γ-LGVO-0.09-NW, electrode area, pulse duration time, electrode thickness; and E_S and E_τ respectively represent the change in the equilibrium voltage and the total voltage change during the current pulse, which can be gained from the GITT steps. The average D_{Li} value of γ-LGVO-0.09-NW is 4.2×10^{-12} cm^2 s^{-1}, in good agreement with that obtained from the CV method. Based on the same GITT method, the average D_{Li} value of γ-LGVO-0.09 remains a reasonably large value of 1.4×10^{-12} cm^2 s^{-1}, but is smaller than that of γ-LGVO-0.09-NW. This result is reasonable since both nanosizing and carbon compositing in γ-LGVO-0.09-NW can increase its apparent Li^+ diffusion coefficients [S10–S13].

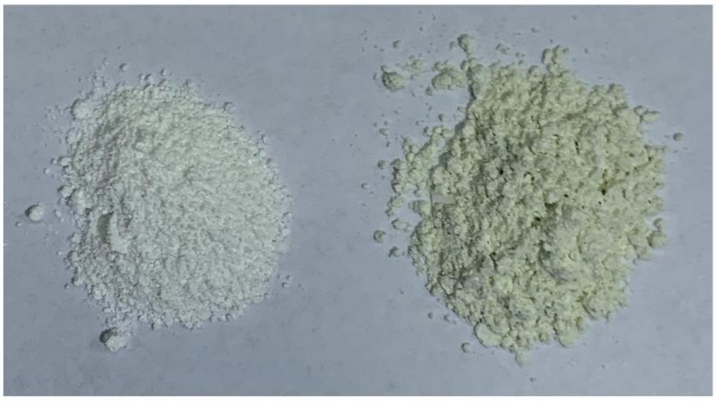

Figure S12. Digital photo of β-Li_3VO_4 (left) and γ-LGVO-0.09 (right) microparticles, revealing white color of β-Li_3VO_4 microparticles and light yellow color of γ-LGVO-0.09 microparticles.

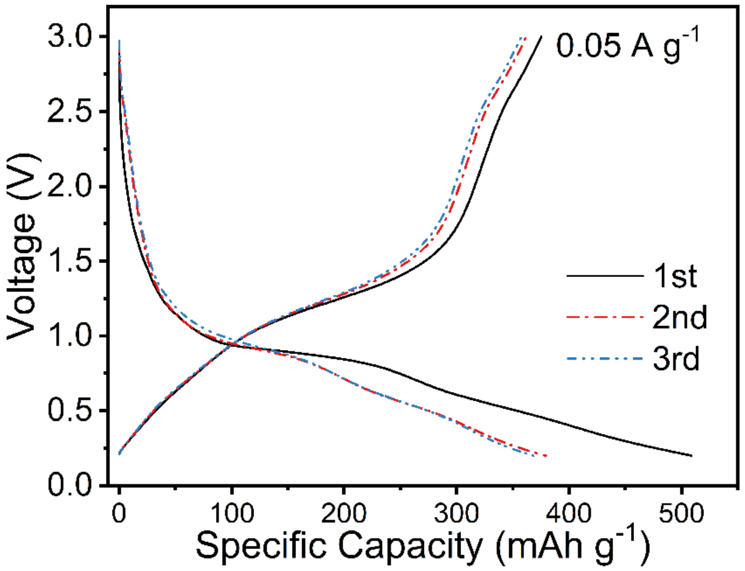

Figure S13. GCD curves of γ-LGVO-0.09-NW/Li half cell for initial three cycles at 0.05 A g^{-1} and 25 ℃.

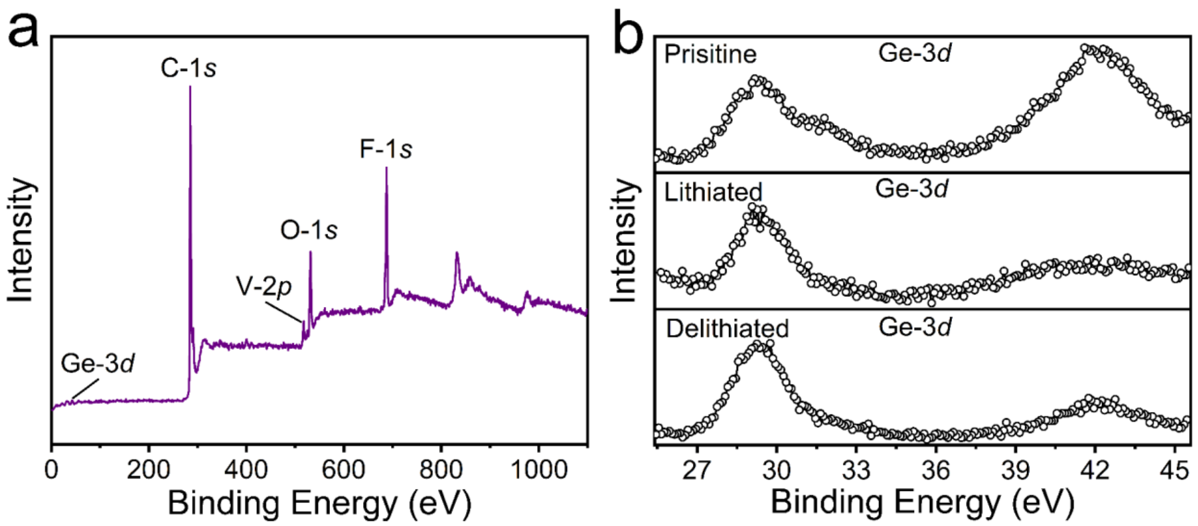

Figure S14. *Ex-situ* XPS spectra of a) pristine γ-LGVO-0.09-NW and b) Ge-3*d* of γ-LGVO-0.09-NW at different states, showing that the chemical valence of germanium is unchanged during lithiation–delithiation.

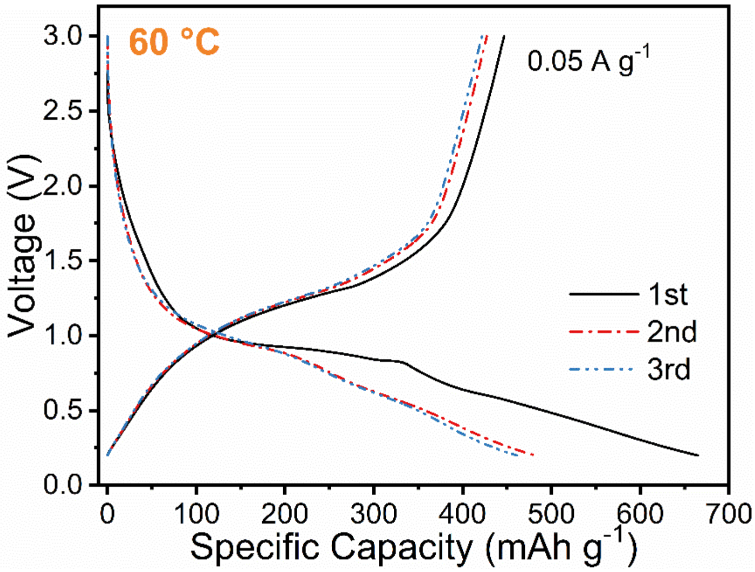

Figure S15. GCD curves of γ-LGVO-0.09-NW/Li half cell for initial three cycles at 0.05 A g^{-1} and 60 °C.

Table S1. Rietveld-refined lattice constants of *γ*-LGVO-0.09, *γ*-LGVO-*x* and *γ*-LSVO-*x* (*x* = 0.06, 0.08 and 0.10).

sample	*a* (Å)	*b* (Å)	*c* (Å)	*V* ($Å^3$)	*R_{wp}
γ-LGVO-0.06	10.9114(4)	6.3459(2)	5.0987(2)	353.05(2)	0.1074
γ-LGVO-0.08	10.9164(3)	6.3484(2)	5. 1026(1)	353.62(2)	0.0557
γ-LGVO-0.09	10.9188(2)	6.3469(1)	5.1091(1)	354.06(1)	0.0573
γ-LGVO-0.10	10.9261(2)	6.3468(1)	5.1123(1)	354.52(1)	0.0951
γ-LSVO-0.06	10.9047(3)	6.3007(2)	5.1551(1)	354.19(2)	0.1002
γ-LSVO-0.08	10.8898(3)	6.3230(1)	5.1122(1)	352.02(2)	0.1018
γ-LSVO-0.10	10.8746(1)	6.3223(1)	5.0865(1)	349.71(1)	0.0732

*R_{wp}: weighted profile residual.

Table S2. Fractional atomic parameters of *γ*-LGVO-0.09 with *Pnma* space group.

atom	site	*x*	*y*	*z*	occupancy
Li(1)	4*c*	0.425772	0.750000	0.183006	1.0000
Li(2)	8*d*	0.168237	0.964880	0.341513	0.7600
Li(3)	8*d*	0.168851	0.085719	0.317402	0.2400
Li(4)	4*c*	0.220075	0.250000	0.880536	0.0450
Li(5)	8*d*	0.152438	0.860489	0.957875	0.0225
Ge	4*c*	0.413553	0.250000	0.329864	0.0900
V	4*c*	0.413553	0.250000	0.329864	0.9100
O(1)	8*d*	0.340018	0.033065	0.215649	1.0000
O(2)	4*c*	0.087503	0.750000	0.166358	1.0000
O(3)	4*c*	0.064049	0.250000	0.275790	1.0000

Table S3. Comparisons of maximum unit-cell-volume change of γ-LGVO-0.09 with intercalating Li^+-storage materials previously reported.

material	maximum volume change (%)	reference
***γ*-LGVO-0.09**	**0.016**	**this work**
$Na_2Ca(VO_3)_4$	0.039	[S14]
γ-$Li_{3.08}Cr_{0.02}Si_{0.09}V_{0.9}O_4$	0.18	[S15]
$Li_4Ti_5O_{12}$	0.2	[S16]
Li_2TiSiO_5	0.37	[S17]
$LiY(MoO_4)_2$	0.53	[S18]
$LiCrTiO_4$	0.69	[S19]
$LiCoO_2$	1.8	[S20]
$LiNi_{0.8}Co_{0.15}Al_{0.05}O_2$	2.13	[S21]
$LiCoMnO_4$	2.2	[S22]
$Li_2PbTi_6O_{14}$	3.41	[S23]
β-Li_3VO_4	4.0	[S24]
$Nb_{18}W_{16}O_{93}$	4.8	[S25]
$LiNi_{1/2}Mn_{3/2}O_4$	5.0	[S26]
$Li_2SrTi_6O_{14}$	5.51	[S23]
VO_2	6.02	[S27]
$LiFePO_4$	6.77	[S28]
$TiNb_2O_7$	7.22	[S29]
$Cu_2Nb_{34}O_{87}$	8.32	[S30]
$Nb_{16}W_5O_{55}$	8.4	[S29]
$ZrNb_{14}O_{37}$	8.56	[S31]
VNb_9O_{25}	8.9	[S32]
$LiMnPO_4$	10.7	[S33]
graphite	13.2	[S34]
$TiNb_{24}O_{62}$	17.5	[S35]

Table S4. Comparisons of electrochemical properties of *γ*-LGVO-0.09-NW with intercalating anode materials previously reported.

material	reversible capacity	cycling stability	reference
***γ*-LGVO-0.09-NW**	**346 mAh g^{-1} at 0.05 A g^{-1}**	**94.8% after 1800 cycles at 2 A g^{-1}**	**this work**
Li_3VO_4 particles	128 mAh g^{-1} at 0.02 A g^{-1}	93.7% after 25 cycles at 0.02 A g^{-1}	[S36]
carbon-coated Li_3VO_4	315 mAh g^{-1} at 0.02 A g^{-1}	84.5% after 50 cycles at 0.02 A g^{-1}	[S37]
carbon-encapsulated Li_3VO_4	450 mAh g^{-1} at 0.1 C	80% after 2000 cycles at 10C	[S38]
Li_3VO_4/Ni	543 mAh g^{-1} at 0.12 A g^{-1}	69.6% after 100 cycles at 0.3C	[S39]
$TiNb_2O_7$ nanoparticles	76 mAh g^{-1} at 10C	~84% after 300 cycles at 1C	[S40]
$TiNb_2O_7$ hierarchical microspheres	100 mAh g^{-1} at 20C	83.5% after 500 cycles at 10C	[S41]
$TiNb_2O_7$ ordered macroporous particles	84 mAh g^{-1} at 20C	~82% after 1000 cycles at 10C	[S42]
$Ti_2Nb_{10}O_{29}$ nanofibers	87 mAh g^{-1} at 30C	49.6% after 2000 cycles at 5 A g^{-1}	[S43]
$Cr_{0.2}Fe_{0.8}Nb_{11}O_{29}$ microparticles	123 mAh g^{-1} at 10C	86.9% after 500 cycles at 10C	[S44]
$GaNb_{11}O_{29}$ microparticles	121 mAh g^{-1} at 10C	87.4% after 1000 cycles at 10C	[S45]
$HfNb_{24}O_{62}$ microparticles	78 mAh g^{-1} at 10C	87.1% after 500 cycles at 10C	[S46]
$W_3Nb_{14}O_{44}$ nanowires	130.6 mAh g^{-1} at 5C	~64% after 1000 cycles at 1 A g^{-1}	[S47]
$Li_4Ti_5O_{12}$ nanorods	135.9 mAh g^{-1} at 10C	61.5% after 1500 cycles at 10C	[S48]
$BaLi_2Ti_6O_{14}$ microparticles	111.7 mAh g^{-1} at 5C	80.7% after 200 cycles at 5C	[S49]

Table S5. Comparisons of apparent Li^+ diffusion coefficient (D_{Li}) of γ-LGVO-0.09-NW with vanadium/niobium/titanium-based anode materials previously reported.

material	D_{Li} ($cm^2\ s^{-1}$)	test technique	reference
**γ-LGVO-0.09-NW (25 °C)**	**3.7×10^{-12}**	**CV**	**this work**
**γ-LGVO-0.09-NW (60 °C)**	**6.8×10^{-12}**	**CV**	**this work**
γ-$Li_{3.08}Cr_{0.02}Si_{0.09}V_{0.9}O_4$ nanowires	1.21×10^{-12}	GITT	[S15]
$Li_{2.98}Cr_{0.04}V_{0.98}O_4$ microparticles	6.21×10^{-13}	GITT	[S50]
Nb_2O_5 nanorods	3.66×10^{-17}	CV	[S51]
$Al_{0.5}Nb_{24.5}O_{62}$ microparticles	2.5×10^{-13}	GITT	[S52]
$GeNb_{18}O_{47}$ nanowires	1.574×10^{-14}	CV	[S53]
$TiNb_2O_7$ nanorods	3.24×10^{-14}	CV	[S54]
$TiNb_6O_{17}$ microparticles	4.28×10^{-14}	CV	[S55]
$MoNb_{12}O_{33}$ microparticles	3.9×10^{-14}	GITT	[S56]
$HfNb_{24}O_{62}$ microparticles	$1.6\sim1.7\times10^{-12}$	GITT	[S46]
$Nb_{16}W_5O_{55}$ microparticles	1.0×10^{-13}	GITT	[S9]
$Nb_{18}W_{16}O_{93}$ nanowires	1.312×10^{-14}	EIS	[S57]
TiO_2/C composite nanospheres	2.2×10^{-15}	CV	[S58]
$Li_4Ti_5O_{12}$ thin films	3.27×10^{-16}	GITT	[S59]

References

[S1] R.D. Shannon, Revised effective ionic radii and systematic studies of interatomic distances in halides and chalcogenides, Acta Cryst. A32 (1976) 751–767.

[S2] M. Nishijima, T. Ootani, Y. Kamimura, T. Sueki, S. Esaki, S. Murai, K. Fujita, K. Tanaka, K. Ohira, Y. Koyama, I. Tanaka, Accelerated discovery of cathode materials with prolonged cycle life for lithium-ion battery, Nat. Commun. 5 (2014) 4553.

[S3] K. Mukai, K. Ariyoshi, T. Ohzuku, Comparative study of Li[CrTi]O_4, Li[$Li_{1/3}Ti_{5/3}$]O_4 and $Li_{1/2}Fe_{1/2}$[$Li_{1/2}Fe_{1/2}$Ti]O_4 in non-aqueous lithium cells, J. Power Sources 146 (2005) 213–216.

[S4] T.A. Assefa, A.F. Suzana, L.L. Wu, R.J. Koch, L.X. Li, W. Cha, R.J. Harder, E.S. Bozin, F. Wang, I.K. Robinson, Imaging the phase transformation in single particles of the lithium titanate anode for lithium-ion batteries, ACS Appl. Energy Mater. 4 (2021) 111–118.

[S5] K.M. Colbow, J.R. Dahn, R.R. Haering, Structure and electrochemistry of the spinel oxides $LiTi_2O_4$ and $Li_{4/3}Ti_{5/3}O_4$, J. Power Sources 26 (1989) 397–402.

[S6] C. Lv, C. Lin, X. Zhao, Rational design and synthesis of nickel niobium oxide with high-rate capability and cycling stability in a wide temperature range, Adv. Energy Mater. 12 (2022) 2102550.

[S7] X. Lou, Q. Fu, J. Xu, C. Lin, J. Han, Y. Luo, Y. Chen, X. Fan, J. Li, $GaNb_{11}O_{29}$ Nanowebs as high-performance anode materials for lithium-ion batteries, ACS Appl. Nano Mater. 1 (2018) 183–190.

[S8] A.J. Bard, L.R. Faulkner, Electrochemical Methods: Fundamentals and Applications, second ed., Wiley, New York, 2001.

[S9] J. Kim, S. Park, S. Hwang, W.B. Yoon, Principles and applications of galvanostatic intermittent titration technology for lithium-ion batteries, J. Electrochem. Sci. Technol. 13 (2022) 19–31.

[S10] G. Liu, L. Zhao, R. Sun, W. Chen, M. Hu, M. Liu, X. Duan, T. Zhang, Mesoporous $TiNb_2O_7$ microspheres as high performance anode materials for lithium-ion batteries with high-rate capability and long cycle-life, Electrochim. Acta 259 (2018) 20–27.

[S11] C. Yang, S. Yu, C. Lin, F. Lv, S. Wu, Y. Yang, W. Wang, Z.Z. Zhu, J. Li, N. Wang, S. Guo, $Cr_{0.5}Nb_{24.5}O_{62}$ nanowires with high electronic conductivity for high-rate and long-life lithium-ion storage, ACS Nano 11 (2017) 4217–4224.

[S12] R. Qian, C. Yang, D. Ma, K. Li, T. Feng, J. Feng, J.H. Pan, Robust lithium storage of block copolymer-templated mesoporous $TiNb_2O_7$ and $TiNb_2O_7$@C anodes evaluated in half-cell and full-battery configurations, Electrochim. Acta 379 (2021) 138179.

[S13] C. Lin, L. Hu, C. Cheng, K. Sun, X. Guo, Q. Shao, J. Li, N. Wang, Z. Guo, Nano-$TiNb_2O_7$/carbon nanotubes composite anode for enhanced lithium-ion batteries of electric vehicles, Electrochim. Acta 260 (2018) 65–72.

[S14] L. Yang, G. Liang, H. Cao, S. Ma, X. Liu, X. Li, G. Chen, W. You, C. Lin, R. Che, A new sodium calcium cyclotetravanadate framework: “zero-strain” during large-capacity lithium intercalation, Adv. Funct. Mater. 32 (2022) 2105026.

[S15] G. Liang, L. Yang, Q. Han, G. Chen, C. Lin, Y. Chen, L. Luo, X. Liu, Y. Li, R. Che, Conductive $Li_{3.08}Cr_{0.02}Si_{0.09}V_{0.9}O_4$ anode material: novel “zero-strain” characteristic and superior electrochemical Li^+ Storage, Adv. Energy Mater. 10 (2020) 1904267.

[S16] S. Panero, P. Reale, F. Ronci, B. Scrosati, P. Perfetti, V. Rossi Albertini, Refined, in-situ EDXD structural analysis of the $Li[Li_{1/3}Ti_{5/3}]O_4$ electrode under lithium insertion–extraction, Phys. Chem. Chem. Phys. 3 (2001) 845–847.

[S17] D. He, B. Wang, T. Wu, H. Su, X. Zhang, Y. Ren, G.-L. Xu, Z. Liu, J. Wang, K. Amine, H. Yu, TiO_2 nanocrystal-framed Li_2TiSiO_5 platelets for low-voltage lithium battery anode, Adv. Funct. Mater. 30 (2020) 2001909.

[S18] N. Peng, X. Cheng, H. Yu, H. Zhu, T. Liu, R. Zheng, M. Shui, Y. Xie, J. Shu, $LiY(MoO_4)_2$ nanotubes: novel zero-strain anode for electrochemical energy storage, Energy Storage Mater. 21 (2019) 297–307.

[S19] M. Luo, H. Yu, X. Cheng, H. Zhu, W. Ye, L. Yan, S. Qian, M. Shui, J. Shu, $LiCrTiO_4$ nanowires with the (111) peak evolution during cycling for high-performance lithium ion battery anodes, ACS Sustainable Chem. Eng. 5 (2017) 10580–10587.

[S20] H. Wang, Y.I. Jang, B. Huang, D.R. Sadoway, Y.M. Chiang, TEM study of electrochemical cycling-induced damage and disorder in $LiCoO_2$ cathodes for rechargeable lithium batteries, J. Electrochem. Soc. 146 (1999) 473–480.

[S21] N. Zhang, X. Zhang, E. Shi, S. Zhao, K. Jiang, D. Wang, P. Wang, S. Guo, P. He, H. Zhou, In situ X-ray diffraction and thermal analysis of $LiNi_{0.8}Co_{0.15}Al_{0.05}O_2$ synthesized via co-precipitation method, J. Energy Chem. 27 (2018) 1655–1660.

[S22] K. Ariyoshi, H. Yamamoto, Y. Yamada, High dimensional stability of $LiCoMnO_4$ as positive electrodes operating at high voltage for lithium-ion batteries with a long cycle life, Electrochim. Acta 260 (2018) 498–503.

[S23] S. Qian, H. Yu, L. Yan, P. Li, X. Lin, Y. Wu, N. Long, M. Shui, J. Shu, Complex titanates $Sr_{1-x}Pb_xLi_2Ti_6O_{14}$ ($0 \leq x \leq 1$) as anode materials for high-performance lithium-ion batteries, Electrochim. Acta 212 (2016) 950–957.

[S24] M.E. Arroyo-de Dompablo, P. Tartaj, J.M. Amarilla, U. Amador, Computational investigation of Li insertion in Li_3VO_4, Chem. Mater. 28 (2016) 5643–5651.

[S25] K.J. Griffith, K.M. Wiaderek, G. Cibin, L.E. Marbella, C.P. Grey, Niobium tungsten oxides for high-rate lithium-ion energy storage, Nature 559 (2018) 556–563.

[S26] K. Ariyoshi, Y. Iwakoshi, N. Nakayama, T. Ohzuku, Topotactic two-phase reactions of $Li[Ni_{1/2}Mn_{3/2}]O_4$ ($P4_332$) in nonaqueous lithium cells, J. Electrochem. Soc. 151 (2004) A296–A303.

[S27] Q. Liu, G. Tan, P. Wang, S.C. Abeyweera, D. Zhang, Y. Rong, Y. A.Wu, J. Lu, C.-J. Sun, Y. Ren, Y. Liu, R.T. Muehleisen, L.B. Guzowski, J. Li, X. Xiao, Y. Sun, Revealing mechanism responsible for structural reversibility of single-crystal VO_2 nanorods upon lithiation/delithiation, Nano Energy 36 (2017) 197–205.

[S28] D. Wang, H. Li, Z. Wang, X. Wu, Y. Sun, X. Huang, L. Chen, New solid-state synthesis routine and mechanism for $LiFePO_4$ using LiF as lithium precursor, J. Solid State Chem. 177 (2004) 4582–4587.

[S29] B. Guo, X. Yu, X.-G. Sun, M. Chi, Z.-A. Qiao, J. Liu, Y.-S. Hu, X.-Q. Yang, J.B. Goodenough, S. Dai, A long-life lithium-ion battery with a highly porous $TiNb_2O_7$ anode for large-scale electrical energy storage, Energy Environ. Sci. 7 (2014) 2220–2226.

[S30] L. Yang, X. Zhu, X. Li, X. Zhao, K. Pei, W. You, X. Li, Y. Chen, C. Lin, R. Che, Conductive copper niobate: superior Li^+-storage capability and novel Li^+-transport mechanism, Adv. Energy Mater. 9 (2019) 1902174.

[S31] Y. Li, R. Zheng, H. Yu, X. Cheng, T. Liu, N. Peng, J. Zhang, M. Shui, J. Shu, Observation of $ZrNb_{14}O_{37}$ nanowires as a lithium container via in situ and ex situ techniques for high-performance lithium-ion batteries, ACS Appl. Mater. Interfaces 11 (2019) 22429–22438.

[S32] S. Qian, H. Yu, L. Yan, H. Zhu, X. Cheng, Y. Xie, N. Long, M. Shui, J. Shu, High-rate long-life pored nanoribbon VNb_9O_{25} built by interconnected ultrafine nanoparticles as anode for lithium-ion batteries, ACS Appl. Mater. Interfaces 9 (2017) 30608–30616.

[S33] G. Li, H. Azuma, M. Tohda, $LiMnPO_4$ as the cathode for lithium batteries, Electrochem. Solid-State Lett. 5 (2002) A135–A137.

[S34] S. Schweidler, L. de Biasi, A. Schiele, P. Hartmann, T. Brezesinski, J. Janek, Volume changes of graphite anodes revisited: a combined operando X-ray diffraction and in situ pressure analysis study, J. Phys. Chem. C 122 (2018) 8829–8835.

[S35] H. Yu, X. Cheng, H. Zhu, R. Zheng, T. Liu, J. Zhang, M. Shui, Y. Xie, J. Shu, Deep insights into kinetics and structural evolution of nitrogen-doped carbon coated $TiNb_{24}O_{62}$ nanowires as high-performance lithium container, Nano Energy 54 (2018) 227–237.

[S36] H. Li, X. Liu, T. Zhai, D. Li, H. Zhou, Li_3VO_4: a promising insertion anode material for lithium-ion batteries, Adv. Energy Mater. 3 (2013) 428–432.

[S37] G. Shao, L. Gan, Y. Ma, H. Li, T. Zhai, Enhancing the performance of Li_3VO_4 by combining nanotechnology and surface carbon coating for lithium ion batteries, J. Mater. Chem. A 3 (2015) 11253–11260.

[S38] C. Zhang, H. Song, C. Liu, Y. Liu, C. Zhang, X. Nan, G. Cao, Fast and reversible Li ion insertion in carbon-encapsulated Li_3VO_4 as anode for lithium-ion battery, Adv. Funct. Mater. 25 (2015) 3497–3504.

[S39] S. Ni, X. Lv, J. Ma, X. Yang, L. Zhang, The fabrication of Li_3VO_4/Ni composite material and its electrochemical performance as anode for Li-ion battery, Electrochim. Acta 130 (2014) 800–804.

[S40] L. Fei, Y. Xu, X. Wu, Y. Li, P. Xie, S. Deng, S. Smirnov, H. Luo, SBA-15 confined synthesis of $TiNb_2O_7$ nanoparticles for lithium-ion batteries, Nanoscale 5 (2013) 11102–11107.

[S41] H. Li, L. Shen, G. Pang, S. Fang, H. Luo, K. Yang, X. Zhang, $TiNb_2O_7$ nanoparticles assembled into hierarchical microspheres as high-rate capability and long-cycle-life anode materials for lithium ion batteries, Nanoscale 7 (2015) 619–624.

[S42] S. Lou, X. Cheng, Y. Zhao, A. Lushington, J. Gao, Q. Li, P. Zuo, B. Wang, Y. Gao, Y. Ma, C. Du, G. Yin, X. Sun, Superior performance of ordered macroporous $TiNb_2O_7$ anodes for lithium ion batteries: understanding from the structural and pseudocapacitive insights on achieving high rate capability, Nano Energy 34 (2017) 15–25.

[S43] D. Pham-Cong, J. Kim, V.T. Tran, S.J. Kim, S.-Y. Jeong, J.-H. Choi, C.R. Cho, Electrochemical behavior of interconnected $Ti_2Nb_{10}O_{29}$ nanoparticles for high-power Li-ion battery anodes, Electrochim. Acta 236 (2017) 451–459.

[S44] X. Lou, Z. Xu, Z. Luo, C. Lin, C. Yang, H. Zhao, P. Zheng, J. Li, N. Wang, Y. Chen, H. Wu, Exploration of $Cr_{0.2}Fe_{0.8}Nb_{11}O_{29}$ as an advanced anode material for lithium-ion batteries of electric vehicles, Electrochim. Acta 245 (2017) 482–488.

[S45] X. Lou, Q. Fu, J. Xu, X. Liu, C. Lin, J. Han, Y. Luo, Y. Chen, X. Fan, J. Li, $GaNb_{11}O_{29}$ nanowebs as high-performance anode materials for lithium-ion batteries, ACS Appl. Nano Mater. 1 (2018) 183–190.

[S46] Q. Fu, H. Cao, G. Liang, L. Luo, Y. Chen, V. Murugadoss, S. Wu, T. Ding, C. Lin, Z. Guo, A highly Li^+-conductive $HfNb_{24}O_{62}$ anode material for superior Li^+ storage, Chem. Commun. 56 (2020) 619–622.

[S47] L. Yan, J. Shu, C. Li, X. Cheng, H. Zhu, H. Yu, C. Zhang, Y. Zheng, Y. Xie, Z. Guo, $W_3Nb_{14}O_{44}$ nanowires: ultrastable lithium storage anode materials for advanced rechargeable batteries, Energy Storage Mater. 16 (2019) 535–544.

[S48] L.J. Xi, H.K. Wang, S.L. Yang, R.G. Ma, Z.G. Lu, C.W. Cao, K.L. Leung, J.Q. Deng, A.L. Rogach, C.Y. Chung, Single-crystalline $Li_4Ti_5O_{12}$ nanorods and their application in high rate capability $Li_4Ti_5O_{12}/LiMn_2O_4$ full cells, J. Power Sources 242 (2013) 222–229.

[S49] X. Lin, S. Qian, H. Yu, L. Yan, P. Li, Y. Wu, N. Long, M. Shui, J. Shu, Advanced $BaLi_2Ti_6O_{14}$ anode fabricated via lithium site substitution by magnesium, ACS Sustainable Chem. Eng. 4 (2016) 4859–4867.

[S50] G. Liang, X. Jin, C. Huang, L. Luo, Y. Chen, C. Lin, Cr^{3+}-doped Li_3VO_4 for enhanced Li^+ storage, Funct. Mater. Lett. 13 (2019) 2050005.

[S51] C. Shi, K. Xiang, Y. Zhu, W. Zhou, X. Chen, H. Chen, Box-implanted Nb_2O_5 nanorods as superior anode materials in lithium ion batteries, Ceram. Int. 43 (2017) 12388–12395.

[S52] Q. Fu, R. Li, X. Zhu, G. Liang, L. Luo, Y. Chen, C. Lin, X.S. Zhao, Design, synthesis and lithium-ion storage capability of $Al_{0.5}Nb_{24.5}O_{62}$, J. Mater. Chem. A 7 (2019) 19862–19871.

[S53] F. Ran, X. Cheng, H. Yu, R. Zheng, T. Liu, X. Li, N. Ren, M. Shui, J. Shu, Nano-structured $GeNb_{18}O_{47}$ as novel anode host with superior lithium storage performance, Electrochim. Acta 282 (2018) 634–641.

[S54] L. Hu, C. Lin, C. Wang, C. Yang, J. Li, Y. Chen, S. Lin, $TiNb_2O_7$ nanorods as a novel anode material for secondary lithium-ion batteries, Funct. Mater. Lett. 9 (2016) 1642004.

[S55] C. Lin, G. Wang, S. Lin, J. Li, L. Lu, $TiNb_6O_{17}$: a new electrode material for lithium-ion batteries, Chem. Commun. 51 (2015) 8970–8973.

[S56] X. Zhu, J. Xu, Y. Luo, Q. Fu, G. Liang, L. Luo, Y. Chen, C. Lin, X.S. Zhao, $MoNb_{12}O_{33}$ as a new anode material for high-capacity, safe, rapid and durable Li^+

storage: structural characteristics, electrochemical properties and working mechanisms, J. Mater. Chem. A 7 (2019) 6522–6532.

[S57] W. Ye, H. Yu, X. Cheng, H. Zhu, R. Zheng, T. Liu, N. Long, M. Shui, J. Shu, Highly efficient lithium container based on non-wadsley-roth structure $Nb_{18}W_{16}O_{93}$ nanowires for electrochemical energy storage, Electrochim. Acta 292 (2018) 331–338.

[S58] W.L. Wang, J.-Y. Park, V.H. Nguyen, E.M. Jin, H.-B. Gu, Hierarchical mesoporous rutile TiO_2/C composite nanospheres as lithium-ion battery anode materials, Ceram. Int. 42 (2016) 598–606.

[S59] F. Wunde, F. Berkemeier, G. Schmitz, Lithium diffusion in sputter-deposited $Li_4Ti_5O_{12}$ thin films, J. Power Sources 215 (2012) 109–115.
